# Recombinant Production and Characterization of Six Ene‐reductases from Penicillium steckii

**DOI:** 10.1002/cbic.202401007

**Published:** 2025-03-24

**Authors:** Pedro H. Damada, Henriette J. Rozeboom, Marco W. Fraaije

**Affiliations:** ^1^ Molecular Enzymology Group Institute of Biomolecular Sciences & Biotechnology University of Groningen Nijenborgh 3 9747 AG Groningen, the Netherlands; ^2^ Laboratório de Química Orgânica e Biocatálise Instituto de Química de São Carlos Universidade de São Paulo Av. João Dagnone, 1100, “Ed. Prof. Wagner Douglas Franco”, Santa Angelina 13563-120 São Carlos SP Brazil

**Keywords:** enzymes, ene-reductase, fungus, *Penicillium steckii*, crystal structures

## Abstract

Fungi, known for their adaptability, are valuable sources of enzymes, making them promising for biocatalyst discovery. This study explored *Penicillium steckii*, primarily recognized for secondary metabolite production, as a source of ene‐reductases (ERs), which reduce α,β‐unsaturated compounds. Eleven ER‐encoding genes were identified, and plasmids for *Escherichia coli* expression were generated. Six ERs (PsOYE1‐6) were successfully produced and purified as soluble FMN‐containing proteins. Sequence analysis classified them into Class II (PsOYE1, PsOYE4, PsOYE6), Class III (PsOYE2, PsOYE3), and Class V (PsOYE5) OYEs. All were active on *p*‐benzoquinone and maleimide, with varying activity on other substrates. Their pH optima ranged from 6 to 7, and they exhibited moderate thermostability (35–50 °C). PsOYE2 was crystallized, and its 2.3 Å structure revealed a stable dimer with a unique active site. PsOYE3, PsOYE4, and PsOYE5 were tested for *R*‐carvone conversion and stereoselectivity, all favouring one diastereomer. These fungal ERs expand the enzymatic toolbox for biocatalysis, emphasizing the need for tailored strategies based on specific applications.

## Graphical abstract

This study explores the catalytic potential of novel ene‐reductases (ERs) from *Penicillium steckii*, a fungal species known for valuable metabolites. Through the recombinant production and purification of six ERs, we identified diverse substrate profiles and moderate stability. Elucidation of the crystal structure of one ER (PsOYE2) revealed unique active site features. This set of recombinant Old Yellow Enzyme (OYE)‐type ERs can be explored for future biocatalytic applications.

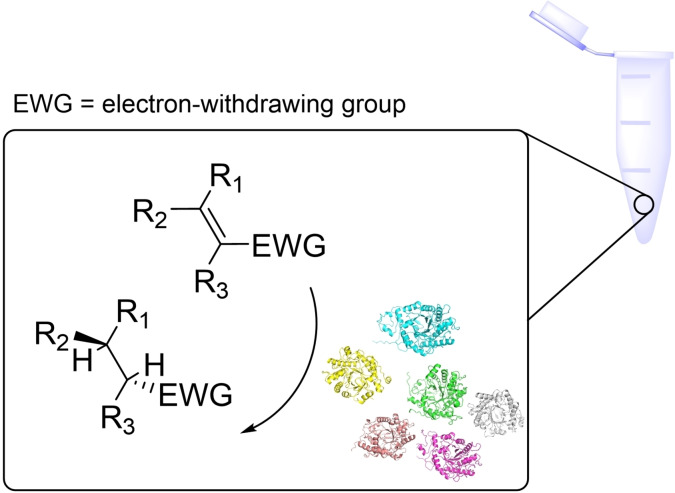

## Introduction

Fungi are highly adaptable organisms capable of thriving in diverse environments, including those with elevated temperatures and high salinity. This adaptability has prompted extensive research into fungi, leading to the discovery of potent bioactive molecules and widely applied enzymes.^[1]^ Advances in fungal genomics have facilitated the identification, characterization, and utilization of these enzymes across various application areas, such as in detergents and functional foods. Furthermore, fungi are gaining recognition as valuable alternatives to traditional chemical catalysts, especially considering their saprophytic nature, which enables them to efficiently break down complex molecules into smaller components.^[2]^

Among fungi, the genus *Penicillium* stands out as one of the most extensively studied and recognized genera. This genus has attracted attention primarily for its ability to produce secondary metabolites, many of which find applications as antitumoral and antibiotic agents.[^3^, ^4^, ^5^] *Penicillium steckii* serves as a notable exemplary within this genus. It has been studied due to its prolific production of various metabolites, such as tanzawaic acids, which inhibit bacterial growth.[^6^, ^7^, ^8^] Given its adaptability and its production of essential metabolites, *P. steckii* represents a promising source of enzymes with biocatalytic potential. Ene‐reductases (ERs) catalyze the asymmetric reduction of C=C double bonds in α,β‐unsaturated compounds, where the double bond is conjugated to an electron‐withdrawing group (EWG), such as a carbonyl moiety.[^9^, ^10^, ^11^]

**Figure 1:**
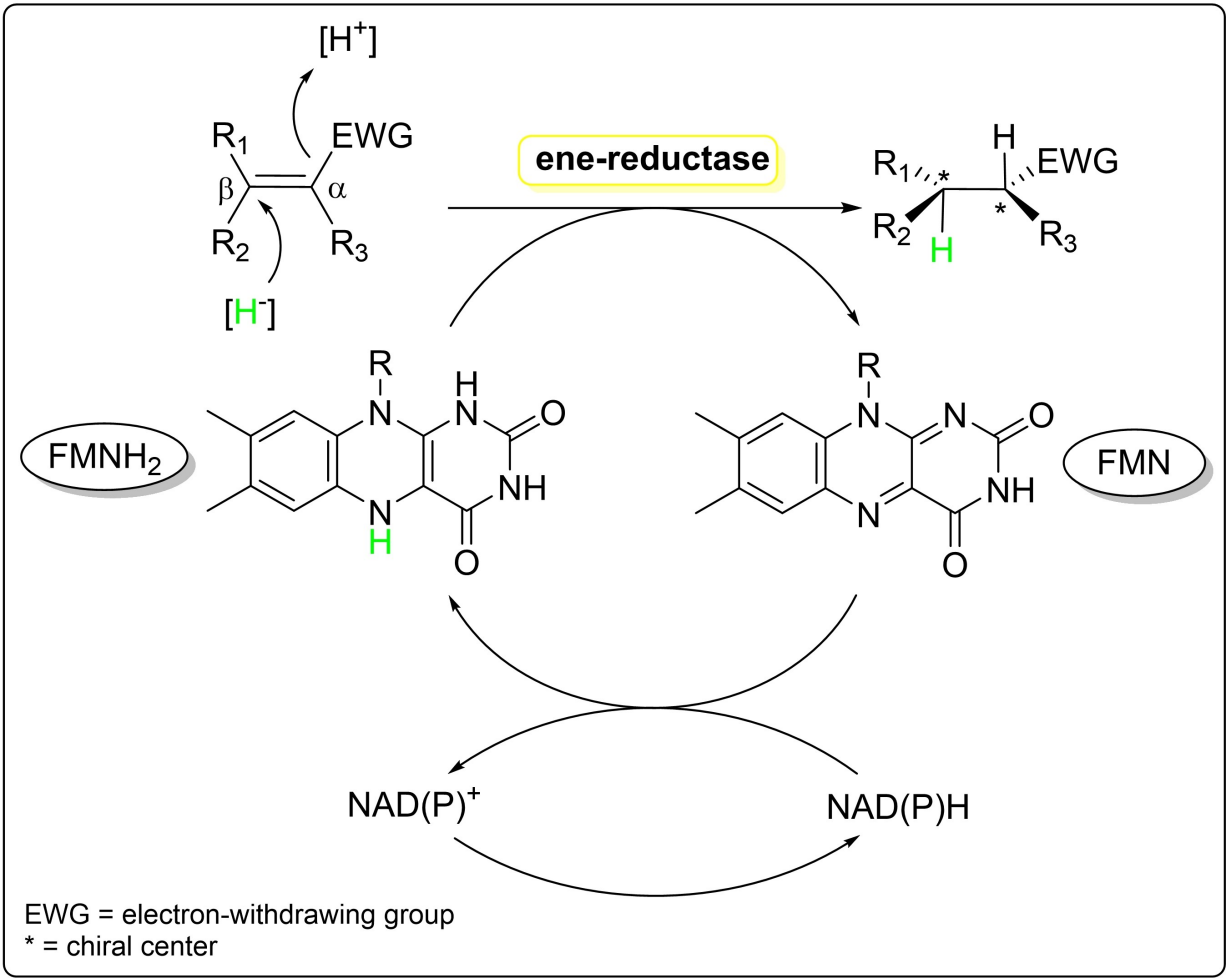
Asymmetric reduction of activated alkenes by ene‐reductase.

The mechanism (Figure 1) can be dissected into two half‐reactions, the first half‐reaction (reductive half‐reaction) begins with the transfer of a hydride from NAD(P)H to the ER‐bound flavin cofactor, FMN. In the next half‐reaction (oxidative half‐reaction), the reduced flavin transfers a hydride ion to the β‐carbon (the carbon next to the EWG). This is accompanied by a proton transfer to the substrate coming from a tyrosine residue in the active site, being transferred to the α‐carbon. This mechanism follows a ping‐pong‐type pattern, resulting in a *trans*‐hydrogenation process that has the potential to create chiral centers.[^12^, ^13^, ^14^] This feature has been the main reason why ERs have become popular biocatalytic tools for the preparation of enantiopure compounds. In recent years, it has been shown that they can also be used for catalyzing oxidations^[15]^ and new‐to‐nature reactions using light.^[16]^

The most extensive ER family is referred to as the Old Yellow Enzyme (OYE) family. The OYE family was sub‐divided into ‘classical’ and ‘thermophilic’ according to the sequence length (thermophilic OYEs having shorter sequences) and the growth conditions of the originating microorganism. Currently, with more representatives, the classification has been adapted. Based on sequence features, OYEs have been categorized into six distinct classes.^[17]^ Representatives of Classes I, II, and III have been the most intensely characterized, and are from bacterial, fungal, and plant origin.^[18]^ Among all enzymes, two ERs have played essential roles in enhancing the understanding of OYEs. OYE1, the first ever described ER,^[19]^ is a Class II OYE that was isolated from the yeast *Saccharomyces pastorianus*. It has been extensively studied since its discovery, which has resulted in insights into its structural and mechanistic details. Studies on OYE1 revealed among others that it adopts a TIM barrel fold and that it can accept a wide range of substrates. Intriguingly, the physiological role of the yeast enzyme is still unclear. Another well‐studied OYE‐type ER is YqjM, which belongs to Class III OYEs. It is a bacterial OYE being part of the proteome of *Bacillus subtilis*. Elucidation of its crystal structure and other characteristics revealed that the OYE family harbors ERs with significantly different active site architectures and quaternary structures.^[20]^ For YqjM, it was shown that an arginine residue from one monomer stretches into the active site of the adjacent monomer, forming a so‐called “arginine finger”. This is a distinctive characteristic, contributing to the formation of a dimeric structure.[^21^, ^22^] OYEs from other classes have been poorly described in the literature. For Class V OYEs, a crystal structure of a fungal representative, ArOYE6 from *Ascochyta rabiei*, has been reported and revealed a somewhat different FMN binding mode and active site pocket when compared with other OYE structures.^[23]^ It shows that exploring other OYEs may bring new insights and will broaden the collection of ER biocatalysts that can be employed for applications.^[24]^

In this study, we focused on a specific fungus, *P. steckii*, for the discovery of novel OYE‐type ERs to broaden the biocatalytic toolbox of ERs. Even though the first OYE (OYE1) was isolated from a fungus, most described OYEs originate from bacteria, leaving fungi underexplored. Analysis of the genome of *P. steckii* revealed a large number of putative OYE‐encoding genes. Production and subsequent characterization will reveal their biocatalytic potential and may also contribute to our understanding of the molecular functioning and physiological role of these enzymes in *P. steckii* and other fungi.

## Results and Discussion

### Identification and Sequence Analyses of new Putative ERs

**Figure 2:**
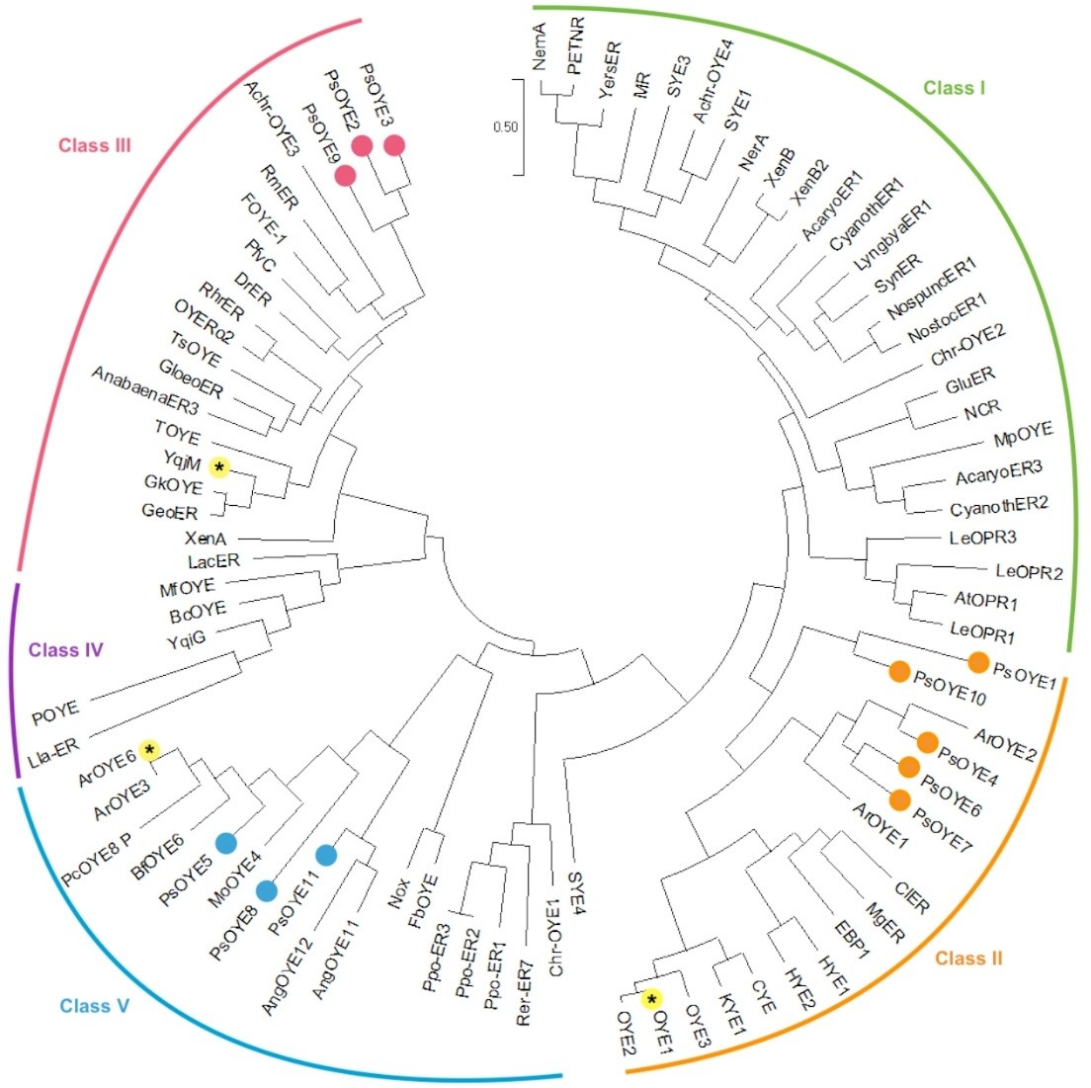
Phylogenic tree constructed using the sequences selected for this work and OYEs described in literature.^[17]^ The analysis involved 85 protein sequences. The enzymes of this study (PsOYE1‐11) were tagged by small dots colored according to the class. The prototype ERs, OYE1, YqjM and ArOYE6, are tagged with an asterisk (*).

Using the BLASTp tool and OYE1 as the input sequence, all the putative ene‐reductases in the predicted proteome of the fungus *P. steckii* were identified, resulting in eleven putative OYE‐type ERs. Compared to other fungal genomes, this is a relatively large number as most fungi were found to harbor 3 to 7 OYE‐type ERs.^[22]^ The proteins were designed as PsOYE1‐PsOYE11 and refer to the following NCBI codes: OQE22947.1, OQE15277.1, OQE15657.1, OQE28785.1, OQE32277.1, OQE13863.1, OQE27379.1, OQE32216.1, OQE18670.1, OQE25206.1, OQE20286.1, respectively. The protein sequences of the eleven proteins were aligned with known ERs to create a phylogenic tree (Figure 2). Expression tests of these eleven *P. steckii* proteins in *E. coli* revealed that PsOYE1‐7 and PsOYE11 could be overexpressed as PTDH‐fused, soluble, and flavin‐containing proteins. However, unfortunately, PsOYE7 and PsOYE11 precipitated during desalting. The remaining proteins were not expressed under the tested conditions, despite varying the expression temperatures (17, 24, and 37 °C). The six expressed proteins, PsOYE1‐6, were categorized into the most recent OYE‐type ERs classification:^[15]^ PsOYE1, PsOYE4, and PsOYE6 were classified as Class II, while PsOYE2 and PsOYE3 were Class III representatives, and PsOYE5 belongs to the poorly explored Class V OYE‐type ERs. The proteins that could not be obtained were from Class II (PsOYE7 and PsOYE10), Class III (PsOYE9), and Class V (PsOYE8 and PsOYE11).

**Figure 3:**
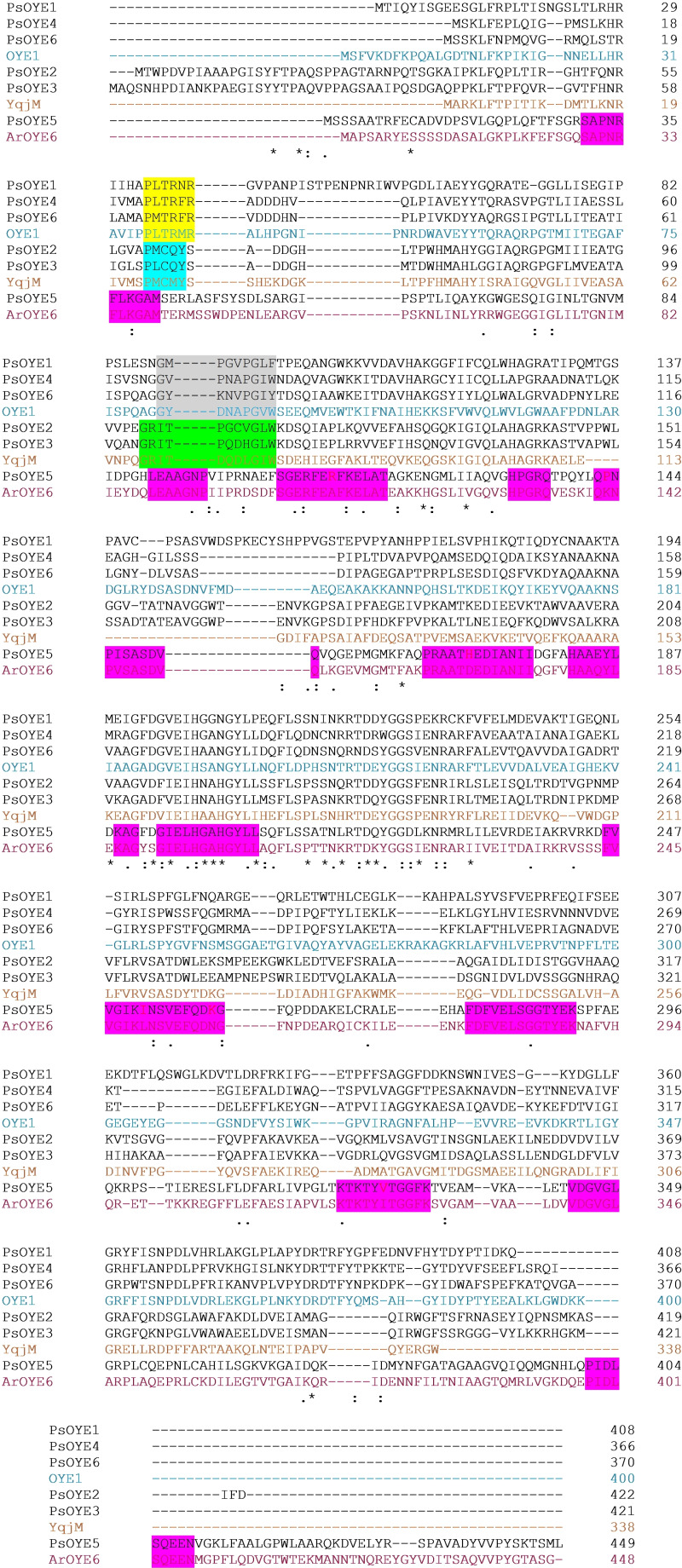
Sequence alignment of OYEs from *P. steckii*, OYE1, YqjM and ArOYE6. The regions highlighted in yellow, and grey represent the patterns indicative for monomers, while the regions highlighted in blue and green represent the dimeric patterns. The conservative regions between ArOYE6 and PsOYE5 are highlighted in purple shade. The symbol (*) denotes full conservation (identical residues across all sequences), (:) indicates strong conservation (similar residues with high chemical similarity), and (.) represents weak conservation (similar residues with low chemical similarity).

The alignment between the PsOYE(1‐6) and the representatives of each class (OYE1, YqjM and ArOYE6) is presented in Figure 3. The OYE Class II is known to be heavily populated by fungal OYEs, with OYE1 being the most well‐studied ER. Sequence comparison revealed that the three Class II ERs from *P. steckii* were highly similar to OYE1, sharing 37–42 % sequence identity. PsOYE4 and PsOYE6 shared the highest sequence identity, while PsOYE1 was more distantly related in sequence. Class III OYEs are typically characterized as thermophilic and often isolated from bacteria, including the well‐studied YqjM. Class III OYEs are distinct in having a unique active site architecture and a different oligomeric organization, often being tetrameric.^[25]^ Class II OYEs, in comparison, are mostly monomeric or dimeric. The sequence identity between the YqjM and PsOYE2 was 44 %, while PsOYE3 shares 39 % sequence identity. PsOYE5 shared only 22–23 % sequence identity with YqjM and OYE1. PsOYE5 shows a 60 % sequence identity with ArOYE6, a representative of Class V OYEs, supporting its classification as a Class V enzyme. Several conservative regions between PsOYE5 and ArOYE6 are highlighted in Figure 3. By aligning the sequences of OYE1, YqjM, ArOYE6 and all six obtained OYEs from *P. steckii*, specific differences in sequence features can be easily seen.

Most OYE‐type ERs are known to exist as monomeric or dimeric proteins, and this characteristic can be predicted based on amino acid sequence patterns.^[26]^ Monomeric ERs exhibit the sequence patterns P‐[LM]‐T−R‐X−R and G‐[FYW]‐X(3)‐P−G‐[ILV]‐[FHYW], while dimeric proteins display slightly different patterns (P−M‐C‐[MQ]‐Y, and G−R‐I‐[TS]‐X(4)‐G−I‐W). The two sequence patterns reflect the structures of two loops: the β1 and β2 loops, respectively. Based on this analysis, the fungal Class II OYEs are expected to be monomeric proteins with the possibility of forming dimers, similar to OYE1 (Figure 3, yellow and grey shades). The sequences of PsOYE2 and PsOYE3 suggest that they are dimeric proteins, while larger oligomers cannot be excluded (Figure 3, blue and green shades). For PsOYE5, no such sequence patterns could be identified, which makes it impossible to predict its oligomeric state.

*AlphaFold2* v2.3.1 was employed to predict the monomeric structures of the ERs. As expected, all predicted structures adopt the typical TIM barrel fold, with a short β‐hairpin lid close to the N‐terminus (Figure S1).[^23^, ^26^, ^27^, ^28^] While the structures display similar overall folds, they also reveal many structural differences.

### Production and Purification of Fungal ERs

PsOYE1‐6, fused to His‐tagged PTDH, were expressed and purified. The enzymes were obtained as soluble, yellow‐colored proteins. The color confirmed the enzymes′ capacity to bind a flavin cofactor. Successful purification was verified by SDS‐PAGE analysis (Figure S2). The observed molecular weights of the fusion proteins were in accordance with the expected molecular weights (85.3 kDa for PsOYE1, 85.0 kDa for PsOYE2, 85.7 kDa for PsOYE3, 80.0 kDa for PsOYE4, 88.7 kDa for PsOYE5 and, 80.5 kDa for PsOYE6). Concentrated purified enzyme samples were stored at −70 °C in 50 mM Tris‐HCl (pH 7.5). All protein samples were flash‐frozen in liquid nitrogen before storage.

UV‐Vis absorbance spectra for all six purified enzymes were obtained (Figure S3), and each displayed two absorbance maxima at around 380 and 460 nm (Table S1), which are typical of a flavin cofactor. The small differences in the position and shape of these absorbance maxima were due to the different protein microenvironments around the isoalloxazine moiety of the FMN cofactor for each protein. The individual molar extinction coefficients of all six OYEs were determined after denaturation and comparison with the known molar extinction coefficient of FMN at 446 nm (Table S1). With this procedure, enzyme concentrations could be accurately quantified. Expression and subsequent purification led to reasonably high yields of pure, soluble, and FMN‐bound proteins (Table S1). The highest yields were obtained for PsOYE4 and PsOYE6, both exceeding 100 mg per liter of culture broth. The lowest yield was obtained for PsOYE5: 29 mg L^−1^.

The strategy of fusing some OYEs (PsOYE2, PsOYE3, and PsOYE5) to a His‐tagged SUMO domain was also tested to facilitate the production of tag‐free proteins. The SUMO tag, which enhances solubility, can be cleaved by SUMO protease, allowing the recovery of untagged proteins. These specific enzymes were selected for investigation due to their unique sequence characteristics and their potential to expand knowledge of different OYE classes. Unfortunately, no significant expression of SUMO‐PsOYE3 was achieved. SUMO‐PsOYE2 and SUMO‐PsOYE5 were obtained as soluble, yellow‐colored proteins, but with relatively low yields: 40 mg L^−1^ for PsOYE2 and 19 mg L^−1^ for PsOYE5. Clearly, expression with PTDH as a fusion partner led to better results in terms of yield.

### Substrate Acceptance

Substrates with different EWGs (such as ketones, maleimides, and carboxylic acid) were tested to assess the biocatalytic activity of the putative ERs by measuring the consumption of NADPH. Activity on one or more test compounds was confirmed for all purified ERs (Table 1). Activities were evaluated using both NADH and NADPH to determine cofactor preference. In all cases, higher activities with NADPH were observed. Therefore, more detailed kinetic analyses were performed with NADPH. The activity of each enzyme with oxygen as an electron acceptor was also measured and subtracted from the activity measured for each substrate. All ERs were found to be able to act as NAD(P)H oxidase, albeit with low activities (0.2–0.6 U/mg; see Table 1).

**Table 1:** Specific activity (U mg^−1^) measured for each enzyme using NADPH and NADH as a cofactor. For some substrates, only activities with NADPH are reported.

Enzyme	PsOYE1 (U mg^−1^)	PsOYE2 (U mg^−1^)	PsOYE3 (U mg^−1^)	PsOYE4 (U mg^−1^)	PsOYE5 (U mg^−1^)	PsOYE6 (U mg^−1^)
Cofactor	NADPH/ NADH	NADPH/ NADH	NADPH/ NADH	NADPH/ NADH	NADPH/ NADH	NADPH/ NADH
O_2_	0.2±0.0/ 0.3±0.0	0.2±0.0/ 0.4±0.2	0.2±0.0/ 0.4±0.1	0.5±0.2/ 0.4±0.0	0.3±0.0/ 0.5±0.0	0.6±0.0/ 0.4±0.0
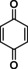	10.9±0.5/ 5.7±1.2	6.7±0.5/ 4.6±0.2	11.7±0.2/ 5.8±0.4	10.3±0.4/ 5.0±0.3	8.6±0.1/ 2.8±0.0	8.4±0.2/ 3.7±0.1
**1**						
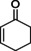	n.d.	2.6±0.9	6.1±0.2	n.d.	1.0±0.0	6.6±0.3
**2**						
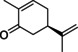	0.2±0.1	1.3±0.0	1.1±0.0	0.1±0.0	0.5±0.0	n.d.
**3**						
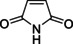	3.2±0.1	2.6±0.1	9.0±0.5	0.6±0.3	0.8±0.0	7.4±0.1
**4**						
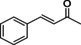	0.6±0.0	n.d.	n.d.	0.3±0.2	n.d.	3.8±0.9
**5**						

None of the enzymes showed activity for all tested substrates. Also, for tiglic aldehyde and cinnamic acid, no activity was found for any of the tested OYEs. Nevertheless, all OYEs presented activity toward *p*‐benzoquinone (**1**) and maleimide (**4**), with a higher activity observed for **1**. PsOYE3 exhibited the highest specific activity toward **1** and **4**: 11.7±0.2 and 9.0±0.5 U mg^−1^, respectively. It is worth noting that PsOYE2 and PsOYE3 belong to the same ER class (Class III); yet PsOYE3 displayed higher specific activity toward *p*‐benzoquinone, while PsOYE2 presented the lowest value. It showed that substrate preference is not directly linked to the type of OYE class.

PsOYE1, PsOYE4, and PsOYE6 activity showed that the type of class does not have to correlate with substrate preference. PsOYE6 exhibits the highest activities toward the tested substrates in comparison with the other two, while PsOYE1 presented limited activity. PsOYE5 exhibited considerable activity with **1** and **4** as well, and the highest activity toward cyclohex‐2‐en‐1‐one (**2**) when compared with the other enzymes.

A previous study demonstrated that different electron‐withdrawing groups (EWGs) influence the reduction process, with reaction times varying depending on the specific EWG. For example, esters tend to prolong the reaction time. Cyclohex‐2‐en‐1‐one (**2**) and maleimide (**5**) are commonly used to evaluate OYE activity, and their conversion rates exceed 90 %, indicating a strong enzymatic preference for these compounds.^[29]^

In this study, *p‐*benzoquinone (**1**) exhibited higher enzymatic activity compared to substrate **2**. Substrates **1** and **5** contain two carbonyl groups, which may enhance the electron‐withdrawing effect and facilitate double‐bond reduction.

The pH optimum for the activity of each PsOYE was determined by measuring activities at pH values ranging from pH 4 to pH 9, using *p*‐benzoquinone as a substrate. It was found that the ERs displayed rather broad pH optima, with maximal activities at pH 6 and 7 (Figure S6). Such pH optima for activity are commonly observed for ERs.[^30^, ^31^]

### Steady State Kinetic Analysis

Since all PsOYEs presented relatively high activities toward *p*‐benzoquinone to form the product, dihydroquinone, this substrate was used to determine the steady state kinetic parameters (*K_M_*, *k_cat_*). The *K_M_* and *k_cat_* values were determined for all PsOYEs (Table 2) by analyzing kinetic data assuming Michaelis‐Menten kinetic behavior (Figures S4 and S5).

**Table 2:** Steady state kinetic parameters (*K_M_* and *k_cat_*) for each enzyme obtained using *p*‐benzoquinone and NADPH as substrates.

Enzyme	Substrate	*K_M_* (mM)	*k_cat_* (s^−1^)	*k*_*cat/*_ *K_M_* (mM^−1^s^−1^)
**PsOYE1**	*p*‐benzoquinone	0.7±0.1	23.4±1.0	33
	NADPH	0.3±0.1	63.0±8.0	200
**PsOYE2**	*p*‐benzoquinone	0.002±0.001	5.0±0.1	2500
	NADPH	0.5±0.1	70.0±10.0	140
**PsOYE3**	*p*‐benzoquinone	0.008±0.001	19.6±1.3	2450
	NADPH	0.2±0.0	64.0±4.0	350
**PsOYE4**	*p*‐benzoquinone	1.1±0.1	28.0±1.0	26
	NADPH	0.3±0.1	53.5±5.0	190
**PsOYE5**	*p*‐benzoquinone	0.9±0.1	23.2±0.9	26
	NADPH	0.5±0.2	70.0±14.0	150
**PsOYE6**	*p*‐benzoquinone	1.1±0.1	25.5±0.9	23
	NADPH	0.3±0.1	53.0±7.0	170

The highest apparent affinity (*K_M_*) for *p*‐benzoquinone was observed for PsOYE2 (0.002 mM), followed by that of PsOYE3 (0.008 mM). These two enzymes belong to the same class and share active site residues, which could explain their highly similar catalytic efficiency for *p*‐benzoquinone. The other enzymes displayed about 100‐fold lower catalytic efficiencies (around 30 mM^−1^ s^−1^), which is mainly due to a relatively high *K_M_* value (around 1 mM). Concerning the hydride donor NADPH, relatively high *K_M_* values were found for all OYEs (Table 2). Using saturating NADPH concentrations, all enzymes reach relatively high rates of ene reduction (around 60 s^−1^).

### Thermostability

The stability of all flavoenzymes was assessed by measuring the apparent melting temperatures (T_m_) using the ThermoFAD procedure.^[32]^ The lowest T_m_ was observed for PsOYE2, which was 37.5 °C, and the highest T_m_ was observed for PsOYE6, at 51.5 °C, indicating its superior stability. PsOYE1 had a T_m_ of 39.0 °C, PsOYE3 had a T_m_ of 44.1 °C, and PsOYE4 had a T_m_ of 47.0 °C. However, for PsOYE5, no fluorescence change was observed, and consequently, the apparent T_m_ could not be estimated using this technique. Then, for PsOYE5, the ThermoFluor^®^ was used, which revealed two unfolding events, at 27.0 and 31.0 °C. This may be due to the unfolding of two distinct protein parts at different temperatures. Further investigations would be required to confirm this hypothesis. It indicates that PsOYE5 is a rather labile enzyme.

PsOYE2 and PsOYE3 belong to Class III OYE‐type ERs. The representatives of this class are often highly thermostable. However, the T_m_ values for these two *P. steckii* ERs were below 45 °C. This was not unexpected since they originated from a mesophilic microorganism. It shows that Class III OYE‐type ERs not only encompass enzymes with considerable thermostability. This has been observed before.[^33^, ^34^] Fungal enzymes are typically not highly thermostable. However, certain thermophilic fungi harbor thermostable enzymes. For instance, CtOYE, an enzyme from *Chaetomium thermophilum*, has a melting temperature of 63 °C.^[35]^

### Structure Determination of PsOYE2

To gain better insights into the structural details of ERs from *P. steckii*, we decided to attempt to obtain crystal structures of selected ERs. For both PsOYE2 and PsOYE5, the SUMO fusion protein was removed to obtain both proteins without any tag (Figure S4). Unfortunately, crystallization trials for PsOYE5 were unsuccessful.

**Figure 4:**
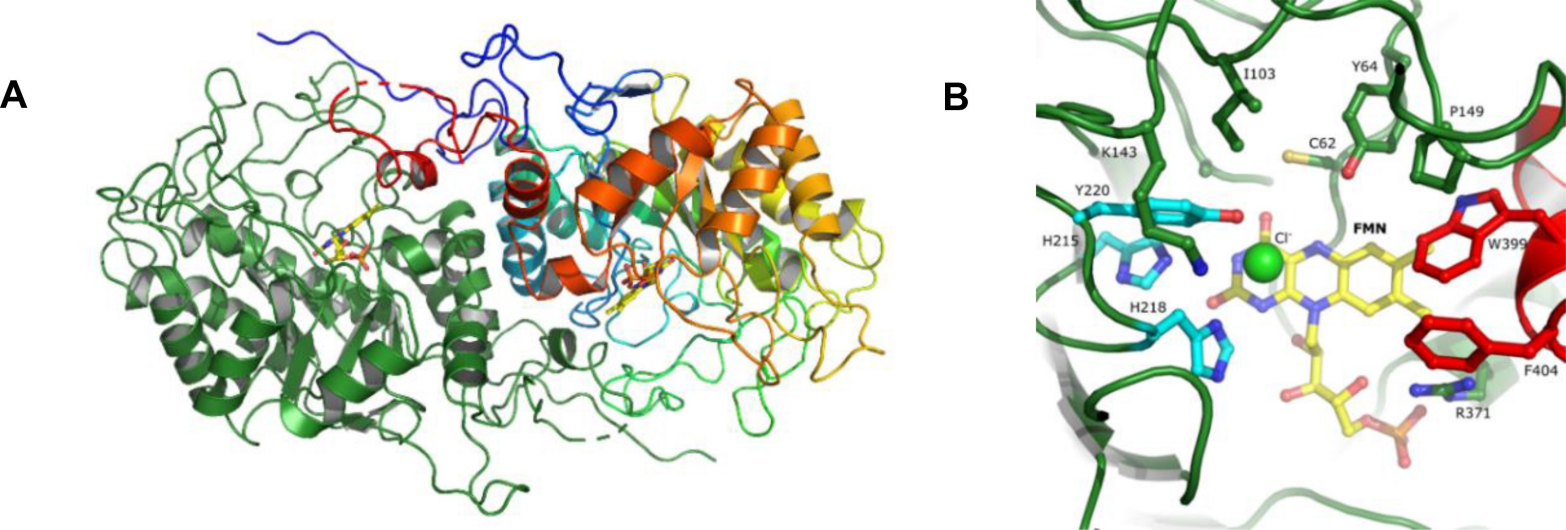
Crystal structure of PsOYE2 from *P. steckii*. (**A**) Ribbon diagram of the overall PsOYE2 dimeric structure in which chain A is colored in green and chain B is rainbow colored (N‐terminus in blue and, C‐terminus in red). The FMN cofactor is shown (represented with carbon, oxygen, nitrogen, and phosphorus atoms in yellow, red, blue, and orange, respectively). (**B**) Zoomed view of the PsOYE2 active site via its entrance. Active site residues are shown as cyan sticks and residues involved in the binding pocket in green. Trp399 and Phe404 are shown on red sticks. A chloride ion is represented as a light green sphere.

For PsOYE2, well‐diffracting crystals were obtained. PsOYE2 crystallized in the monoclinic space group *P*2_1_ with four dimers in the asymmetric unit. The crystal structure was determined at a resolution of 2.3 Å. DLS analysis and size exclusion chromatography already suggested that PsOYE2 forms dimers in solution, with estimated molecular weights of 68 kDa and 78 kDa, respectively. This dimeric oligomerization state aligns with the prediction based on conserved sequence features, vide supra. Moreover, the crystal structure confirmed the presence of a dimer (Figure 4A). PISA analysis (https://www.ebi.ac.uk/pdbe/pisa/) showed a buried surface area of 2710 Å^2^ per interface between monomers (A−B) with a Complexation Significance Score (CSS) of 0.64. The dimer's dimensions were 960×47×53 Å. Nineteen hydrogen bonds and two salt bridges (Arg^375^‐Asp^376^) contributed to the dimerization surface. Although an octameric assembly of the dimers was observed in the crystal, intradimer interactions (A−C) were fewer than interdimer interactions (760 Å^2^ buried surface, CSS=0.13). The closest structural homologues of PsOYE2 determined by a DALI search (http://ekhidna2.biocenter.helsinki.fi/dali/) include: AnOYE8 from *Aspergillus niger* (Z_score_=65.4, 67 % identical on residue level (id), root‐mean‐square deviation (RMSD) of 0.8 Å, PDB 7QFX), BfOYE4 from *Botryotinia fuckeliana* (Z=62.0, 56 % id, RMSD 1.1 Å, PDB 7BLF),^[36]^ *Chloroflexus aggregans* ene‐reductase (Z=54.6, 44 % id, RMSD 1.4 Å, PDB 7O0T^[37]^), OYE from the thermophilic *Ferrovum* sp. JA12 (Z=53.8, 43 % id., RMSD 1.3 Å, PDB 8PUN (not published)), and TsOYE (chromate reductase) from *Thermus scotoductus* (Z=49.2, 46 % id., RMSD 1.2 Å, PDB 3HF3^[38]^). The structure‐based sequence alignment of PsOYE2 with 7BLF and 7QFX is shown in the Supporting Information (Figure S8).

Notably, AnOYE8 and BfOYE4,^[36]^ when compared to PsOYE2, exhibit similar buried surface areas but fewer hydrogen bonds (14 for AnOYE8 and 16 for BnOYE4), along with only one salt bridge.

The enzyme monomeric structure (Figure 4A) includes the residues from 2 to 413. However, most of the eight monomers had a flexible loop (residues 404–406), which were not defined in electron density. Additionally, the C‐termini (414‐422) were not visible. The ER fold consists of eight twisted β‐strands surrounded by eight α‐helices, [(β/α)_8_] or TIM‐barrel structure, as predicted previously using *Alphafold2* and in alignment with other Class III enzymes. A substantial portion of the N‐terminus (44 residues) interacts with the neighboring protomer, creating a tightly bound dimer, which might be a characteristic of fungal enzymes.^[34]^

A small β‐hairpin (45‐47/50‐52) closes the barrel on the N‐terminal side. The C‐terminal region is also involved in dimer formation and shapes the FMN binding pocket of the other monomer. The FMN cofactor, situated on the C‐terminal side of the barrel, has interactions through the phosphate group with the side chain of Arg^371^ and backbone nitrogen atoms of Arg^371^, Gly^370^ and Thr^348^ (of the adjacent protomer). The ribityl part is stabilized by Arg^368^, and the isoalloxazine on the *re* face via hydrophobic contacts with Pro^60^ and Met^61^, and the backbone carbonyl atom of Pro^60^ to N5 and N10. The hydrophobic analogous to Pro^58^ and Met^59^ in AnOYE8 and BfOYE4, confirming a conserved feature in these Class III OYEs.^[36]^ Further interactions of the flavin isoalloxazine ring were observed with side chains of Gln^136^, backbone nitrogen atom of Ala^94^ and sulfur atom Cys^62^ to O4, and backbone nitrogen atom of Cys^62^ to N5.

The “arginine finger”, a distinctive characteristic of Class III ERs, was observed in PsOYE2 being represented by Arg^371^. This residue was not conserved when compared to YqjM (Figure 3), as it appeared in a different position. In place of Arg in comparison to YqjM, PsOYE2 exhibited a Trp, which participates in the substrate binding site and might contribute to form a large hydrophobic substrate site, along with Phe, another residue believed to play a role at the same site. Compared to other enzymes in this class, AnOYE8 and BfOYE4 feature a unique substitution, where the arginine finger is replaced by a bulkier hydrophobic residue, such as tryptophan. The authors suggest that this modification may influence enzymatic activity, which aligns with the restricted substrate specificity observed for AnOYE8. However, this effect was not observed in BfOYE4. The absence of arginine in this position has also been reported in other enzymes, such as XenA.

The entrance to the open active site (Figure 4B) is formed by residues of loops (after β2, β3, β4, and β6) on the C‐terminal side of the barrel and, C‐terminal residues from the adjacent protomer (Trp^399^ and Phe^404^). The binding pocket on the *si* face of the FMN is further shaped by residues Cys^62^, Tyr^64^, Ala^94^, Ile^103^, Lys^143^, Pro^149^, and Arg^371^, in conjunction with the active site residues His^215^, His^218^, and Tyr^220^. Electron density for a molecule larger than a water molecule was observed and tentatively modeled as a chloride ion, present in the crystallization solution. The chloride atom has interactions to the side chain of Tyr^220^ and is at 3.5 Å from C4 A and C10 A of the isoalloxazine ring of FMN. At this position, a formate ion is observed in BfOYE4^[34]^ and a sulfate ion in AnOYE8.^[31]^

Atomic coordinates and experimental structure factor amplitudes were deposited in the Protein Data Bank (PDB) under entry 8S4P.

### Bioconversion

One enzyme from each class was selected for conversion studies using *R*‐carvone, which is a well‐known substrate commonly used to evaluate the stereoselectivity of this enzyme class. Additionally, the reduction of *R*‐carvone is of significant interest due to the versatility of its product, dihydrocarvone, which has applications in various fields.

The enzymes tested were PsOYE4 (Class II), PsOYE3 (Class III), and PsOYE5 (Class V). Although activity was low for PsOYE4 and PsOYE5, these enzymes were included to evaluate the extent of conversion over an extended reaction time. All enzymes successfully reduced *R*‐carvone after 24 hours of reaction (Table 3). For PsOYE3, the reaction reached completion, whereas for PsOYE4 and PsOYE5, residual *R*‐carvone was still detectable. This indicates a slower reaction, consistent with the low activity observed in the standard activity assay. It is important to note that a NADPH recycling system was added to maintain enzyme activity during the conversion. The chromatograms are presented at the Supplementary Material (Figure S9).

**Table 3:** Conversion and *d.e*. values obtained upon conversion of *R*‐carvone.

Enzyme	Class	Conversion (%)^[a]^	*d.e*. (%)/conf.^[b]^
**PsOYE4**	II	71.1	98/ (*R*,*R*)
**PsOYE3**	III	>99	>99/ (*R*,*R*)
**PsOYE5**	V	66.2	>99/ (*R*,*R*)

[a] Conversion determined by GCMS analysis, [b] config.=absolute configuration.

Regarding stereoselectivity, PsOYE3 and PsOYE5 exclusively produced (*R*,*R*)‐dihydrocarvone. PsOYE4 generated a mixture of diastereomers, with (*R*,*R*)‐dihydrocarvone as the major product and a minor fraction of (*S*,*R*)‐dihydrocarvone, resulting in a diastereomeric excess (*d.e*.) of 98 %. In fact, most OYEs described in the literature have shown a preference for the *R,R* stereoisomer.^[34]^

## Conclusions

Out of eleven identified putative OYE‐type ERs from *P. steckii*, six OYEs were successfully expressed as soluble and active flavoenzymes, when using a bacterial expression host. The enzymes expressed fused to PTDH displayed higher yields compared to those expressed with SUMO as N‐terminal tag, showing that, in this case, PTDH has a better effect on expression levels. Based on their sequences analysis, the ERs were classified into three distinct classes: PsOYE1, PsOYE4 and PsOYE6 being Class II OYEs, PsOYE2 and PsOYE3 being Class III OYEs, and PsOYE5 being Class V OYE. NADPH was found to be the preferred electron donor for all the enzymes. All ERs exhibited the ability to reduce one or more of the tested substrates, with high activities observed for *p*‐benzoquinone as substrate. Among the classes, the enzymes in Class III demonstrated greater catalytic efficiency toward this compound. Notably, all OYEs exhibited melting temperatures below 50 °C, indicative of their moderate thermostability. For PsOYE2, the crystal structure could be determined at a resolution of 2.3 Å. The structure includes a bound FMN cofactor and reveals the details of its active site.

Using *R*‐carvone as a substrate, the stereoselectivity of three enzymes – PsOYE3 (Class III), PsOYE4 (Class II), and PsOYE5 (Class V) – were evaluated. PsOYE3 achieved complete conversion with high stereoselectivity. In summary, this study provides access to a set of new and active OYE‐type fungal ERs that can be produced as recombinant proteins. They cover three out of the six OYE classes and enlarge the biocatalytic toolbox of available OYE‐type ER.

## Conflict of Interests

The authors declare no conflict of interest.

1

## Supporting information

As a service to our authors and readers, this journal provides supporting information supplied by the authors. Such materials are peer reviewed and may be re‐organized for online delivery, but are not copy‐edited or typeset. Technical support issues arising from supporting information (other than missing files) should be addressed to the authors.

Supporting Information

## Data Availability

The data that support the findings of this study are available from the corresponding author upon reasonable request.
