# Supplementary material for: Recombinant Production and Characterization of Six Ene‐reductases from Penicillium steckii
Source: Chembiochem. 2025 Mar 24;26(7):e202401007. doi: 10.1002/cbic.202401007 (PMC12002099; doi:10.1002/cbic.202401007)
Supplement: Supplementary file 1 — Supporting Information [file CBIC-26-e202401007-s001.pdf]

ChemBioChem

Supporting Information

Recombinant Production and Characterization of Six Ene-reductases from Penicillium steckii

Pedro H. Damada, Henriette J. Rozeboom, and Marco W. Fraaije*

Supporting Information

Recombinant production and characterization of six ene-reductases from *Penicillium steckii*

Pedro H. Damada[1,2], Henriette J. Rozeboom[1], Marco W. Fraaije[1*]

[1]Molecular Enzymology Group, Groningen Institute of Biomolecular Sciences & Biotechnology, University of Groningen, Nijenborgh 3, 9747 AG Groningen, Netherlands.

[2] Laboratório de Química Orgânica e Biocatálise, Instituto de Química de São Carlos, Universidade de São Paulo, Av. João Dagnone, 1100, "Ed. Prof. Wagner Douglas Franco", Santa Angelina, 13563-120, São Carlos, SP, Brazil.

List of Content

Experimental Section

Chemicals, strains, and enzymes.......... 2

Bioinformatic analysis 2

Plasmid construction.......... 4

Expression and purification.......... 4

Spectral analysis 5

Enzyme activity analysis.......... 7

Analysis of thermostability 9

Crystallization and structure determination 9

Bioconversion.......... 12

Supporting Figure

Figure S1. Structure predicted for the enzymes PsOYE1 (**A**), PsOYE2 (**B**), PsOYE3 (**C**), PsOYE4 (**D**), PsOYE5 (**E**) and PsOYE6 (**F**) by the software *AlphaFold2*and displayed by *PyMOL*. 4

Figure S2. SDS-PAGE analysis of purified enzymes. The first lane shows the protein ladder and the lanes the enzymes in the following order: PsOYE1, PsOYE2, PsOYE3, PsOYE4, PsOYE5 and PsOYE6.......... 5

Figure S3. Absorbance spectra for PsOYE1 (**A**), PsOYE2 (**B**), PsOYE3 (**C**), PsOYE4 (**D**), PsOYE5 (**E**) and PsOYE6 (**F**), native (–) and denatured (--).......... 6

Figure S4. Michaelis-Menten curves obtained by the software GraphPad Prism for measurement of kinetic parameters (K_M and k_{cat}) for the enzymes PsOYE1, PsOYE2, PsOYE3, PsOYE4, PsOYE5 and PsOYE6.for *p*-benzoquinone The kinetic parameters were calculated by analyzing the consumption of NADPH at 340 nm. 7

Figure S5. Michaelis-Menten curves obtained by the software GraphPad Prism for measurement of kinetic parameters (K_M and k_{cat}) for the enzymes PsOYE1, PsOYE2, PsOYE3, PsOYE4, PsOYE5 and PsOYE6 for NADPH using *p*-benzoquinone in fixed concentration (1mM). The kinetic parameters were calculated by analyzing the consumption of NADPH at 340nm. 8

Figure S6. pH optima for activity for PsOYE1 (**A**), PsOYE2 (**B**), PsOYE3 (**C**), PsOYE4 (**D**), PsOYE5 (**E**) and PsOYE6 (**F**). 9

Figure S7. SDS-PAGE analysis of the purified enzymes (PsOYE2 and PsOYE5) after removing SUMO. The first lane shows the protein ladder, and the followings lanes correspond to washing solution (WS), elution solution 1 and 2 (ES1/2) and PsOYE2 and PsOYE5. The SUMO protein in the gel was circled. 10

Figure S8. Structure-based sequence alignment of PsOYE2 and 7BLF (OYE4 from *Botryotinia fuckeliana* (BfOYE4)) and 7QFX (OYE8 from *Aspergillus niger* (AnOYE8)). The alignment was made with SSM (ebi.ac.uk/msd-srv/ssm/). The secondary structure elements (top line) are from the crystal structure of PsOYE2. Identical residues have a red background, similar residues have a red color. The active site residues are in cyan and Phe^{404} and Trp^{399} are in orange. The figure was created with *ESPrip* 12

Figure S9. Chromatograms and mass spectra obtained during the bioconversion of *R*-carvone (**a**) into (2*R*,5*R*)-dihydrocarvone (**b**) and (2*S*,5*R*)-dihydrocarvone (**c**) are presented as follows: Chromatogram (**A**) for the reaction with PsOYE3 and mass spectrum related to the peak of compound **b** (**A1**); chromatogram (**B**) for the reaction with PsOYE4 and mass spectra for the peaks of the compounds: **a** (**B1**), **b** (**B2**), and **c** (**B3**); chromatogram (**C**) for the reaction with PsOYE5 and mass spectra for the peaks of the compounds: **a** (**C1**), and **b** (**C2**). Mesitylene was used as an internal standard (RT = 8.6 min). 14

Figure S10. Chromatogram illustrating the composition of commercial dihydrocarvone, consisting of a mixture of isomers with varying concentrations. The concentration of *R,R*-dihydrocarvone (**a**) is higher than that of *S,R*-dihydrocarvone (**b**).......... 14

Supporting Table

Table S1. List of putative and obtained ene-reductases described in the literature, with the enzymes' source (bacteria, fungi, algae and plants) and the NCBI code access. 2

Table S2. Molar extinction coefficients and yields of purified proteins. 6

Table S3. Data collection and refinement statistics for PsOYE2. 11

Experimental Section

Chemicals, strains, and enzymes

T4 ligase and the restriction enzyme BsaI were purchased from New England Biolabs. *E. coli* NEB 10-beta (NEB, Ipswich, MA, USA) was used as the host for cloning and protein expression. Tiglic aldehyde (98%), *R*-carvone (98%), dihydrocarvone (98% - mixture of isomers), cinnamic acid (97%), cyclohex-2-en-1-one (98%), maleimide (99%), methyl cinnamate (99%), *p*-benzoquinone (98%), *trans*-4-phenyl-but-3-en-2-one (99%) and dimethyl sulfoxide (DMSO, 99%) were obtained from *Sigma-Aldrich* (St. Louis, MO, USA).

Bioinformatic analysis

Using OYE1 as a query, and selecting *P. steckii* as a filter, genes putatively encoding ERs were identified using BLASTp (protein-protein Basic Local Alignment Search Tool) from NCBI (National Center for Biotechnology Information). The search resulted in 11 putative OYE-ERs. To construct a phylogenetic tree, the sequences of the 11 proteins were aligned with those of previously identified ERs [1] (Table S1). The phylogenetic tree was generated using the software *MEGA* v.11 [2], employing the maximum likelihood method based on the JTT matrix-based model [3]. The evolutionary distances were computed using the Poisson correction method [4] and are in the units of the number of amino acid substitutions per site. All ambiguous positions were removed for each sequence pair (pairwise deletion option). The sequences used for the tree were aligned prior to tree construction by using *Clustal-Omega* within *MEGA.*

Table S1. List of putative and obtained ene-reductases described in the literature, with the enzymes' source (bacteria, fungi, algae and plants) and the NCBI code access.

Enzyme	Microorganism origin	NCBI code access
**AcaryoER1**	*Acaryochloris marina MBIC11017*	ABW29811
**AcaryoER3**	*Acaryochloris marina MBIC11017*	ABW32756.1
**Achr-OYE3**	*Achromobacter* sp. *JA81*	AFK73187
**Achr-OYE4**	*Achromobacter* sp. *JA81*	AFK73188
**AnabaenaER3**	*Trichormus variabilis ATCC 29413*	ABA25236
**AngOYE11**	*Aspergillus niger CBS 513.88*	XP_001390672
**AngOYE12**	*Aspergillus niger CBS 513.88*	XP_001395504
**ArOYE1**	*Ascochyta rabiei*	AHL17019
**ArOYE2**	*Ascochyta rabiei*	AHL17020
**ArOYE3**	*Ascochyta rabiei*	AHL17024
**ArOYE6**	*Ascochyta rabiei*	AHL17024
**AtOPR1**	*Arabidopsis thaliana*	NP_177794
**BcOYE**	*Bacillus cereus*	PFE58769
**BfOYE6**	*Botrytis cinerea B05.10*	XP_001547575
**Chr-OYE1**	*Chryseobacterium* sp. *CA49*	ALE60336
**Chr-OYE2**	*Chryseobacterium* sp. *CA49*	ALE60337
**ClER**	*Clavispora lusitaniae ATCC*	EEQ40235
**CyanothER1**	*Rippkaea orientalis PCC 8801*	ACK64210
**CyanothER2**	*Rippkaea orientalis PCC 8801*	ACK65723
**CYE**	*Kluyveromyces marxianus*	BAD24850
**DrER**	*Deinococcus radiodurans*	AAF11740
**EBP1**	*Candida albicans*	AAA18013
**FbOYE**	*Frankiales bacterium*	TAL24079
**FOYE-1**	*Ferrovum* sp. *JA12*	KRH78075
**GeoER**	*Geobacillus sp*	BAO37313

**GkOYE**	*Geobacillus kaustophilus HTA426*	BAD76617
**GloeoER**	*Gloeobacter violaceus PCC 7421*	BAC91769
**GluER**	*Gluconobacter oxydans 621H*	AAW60280
**HYE1**	*Ogataea angusta*	AAN09952
**HYE2**	*Ogataea angusta*	AAN09953
**KYE1**	*Kluyveromyces lactis*	AAA98815
**LacER**	*Lactobacillus paracasei*	ADK19581
**LeOPR1**	*Solanum lycopersicum*	NP_001234781
**LeOPR2**	*Solanum lycopersicum*	NP_001233868
**LeOPR3**	*Solanum lycopersicum*	NP_001233873
**Lla-ER**	*Lactococcus lactis*	QBR53094
**LyngbyaER1**	*Lyngbya* sp. *PCC 8106*	EAW37813
**MfOYE**	*Mumia flava*	KHL17902
**MgER**	*Meyerozyma guilliermondii ATCC 6260*	EDK41665
**MoOYE4**	*Pyricularia oryzae 70-15*	XP_003720070
**MpOYE**	*Micromonas pusilla CCMP1545*	XP_003062818
**MR**	*Pseudomonas putida*	AAC43569
**NCR**	*Zymomonas mobilis*	AAV90509
**NemA**	*Escherichia coli*	BAA13186
**NerA**	*Agrobacterium tumefaciens*	CAA74280
**NospuncER1**	*Nostoc punctiforme PCC 73102*	ACC84535
**NostocER1**	*Nostoc* sp. *PCC 7120*	BAB73564
**Nox**	*Rhodococcus erythropolis*	ALG03744
**OYE1**	*Saccharomyces pastorianus*	Q02899
**OYE2**	*Saccharomyces cerevisiae S288C*	Q03558
**OYE3**	*Saccharomyces cerevisiae S288C*	P41816
**OYERo2**	*Rhodococcus opacus 1CP*	ALL54975
**PcOYE8**	*Penicillium rubens Wisconsin 54-1255*	XP_002560444
**PETNR**	*Enterobacter cloacae*	AAB38683
**PfvC**	*Arthrobacter* sp.	AFF18622
**POYE**	*Propionibacterium* sp.	SPF68004
**Ppo-ER1**	*Paenibacillus polymyxa CR1*	AHC19521
**Ppo-ER2**	*Paenibacillus polymyxa CR1*	AIW41616
**Ppo-ER3**	*Paenibacillus polymyxa*	QBR53093
**Rer-ER7**	*Rhodococcus erythropolis*	QBR53095
**RhrER**	*Rhodococcus rhodochrous ATCC 17895*	AMD82542
**RmER**	*Cupriavidus metallidurans CH34*	ABF11721
**SYE1**	*Shewanella oneidensis MR-1*	AAN55488
**SYE3**	*Shewanella oneidensis MR-1*	AAN57126
**SYE4**	*Shewanella oneidensis MR-1*	AAN56390
**SynER**	*Synechococcus elongatus PCC 7942*	ABB56505
**TOYE**	*Thermoanaerobacter pseudethanolicus*	ABY93685
**TsOYE**	*Thermus scotoductus SA-01*	CAP16804
**XenA**	*Pseudomonas putida*	AAF02538
**XenB**	*Pseudomonas fluorescens*	AAF02539
**XenB2**	*Pseudomonas putida*	AGS77941
**YersER**	*Yersinia bercovieri*	WP_032896199
**YqiG**	*Bacillus subtilis*	QBR53092
**YqjM**	*Bacillus subtilis*	BAA12619

AlphaFold2 v2.3.1 was employed to predict the monomeric structures of the ERs, while *PyMOL v2.5.2*, to display the structures predicted as shown by Figure S1.

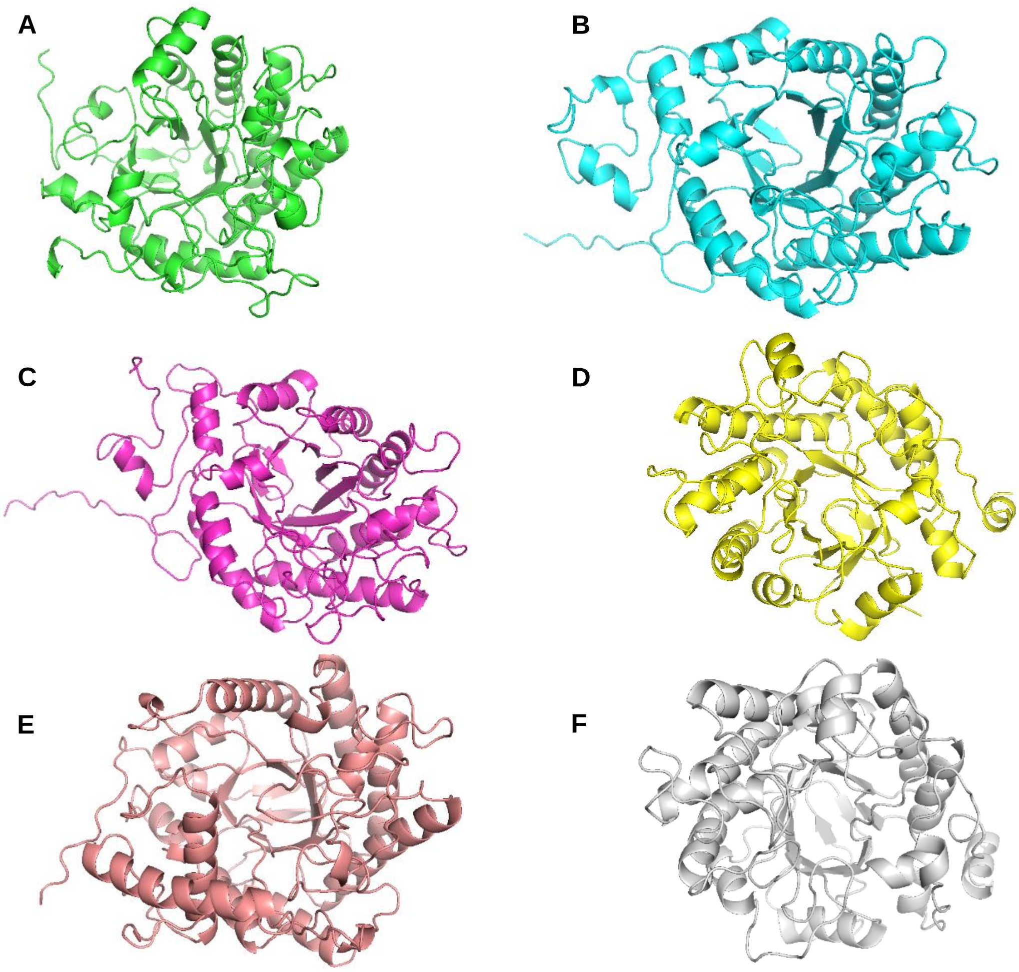

Figure S1. Structure predicted for the enzymes PsOYE1 (**A**), PsOYE2 (**B**), PsOYE3 (**C**), PsOYE4 (**D**), PsOYE5 (**E**) and PsOYE6 (**F**) by the software *AlphaFold2*and displayed by *PyMOL*.

Plasmid construction

The genes, optimized for expression in *E. coli*, were synthesized by *Twist Bioscience* (San Francisco, CA, USA). These DNA fragments were cloned into the vectors pCRE [5] and pBAD-SUMO, using the Golden Gate method [6]. When the vector pBAD-SUMO was used, the protein of interest was expressed with an N-terminal His-tagged SUMO as fusion protein. When the vector pCRE was employed, the protein was expressed with an N-terminal His-tagged phosphite dehydrogenase (PTDH) as fusion protein. SUMO is a protein that often boosts expression of soluble protein, and it can be cleaved from the target protein by using a dedicated SUMO protease. The latter enables the isolation of native protein, suited for crystallographic studies. On the other hand, PTDH facilitates the regeneration of the reduced nicotinamide cofactor (NADH or NADPH) required by the ERs using phosphite as cheap cosubstrate. Additionally, PTDH may also contribute to the expression of soluble protein [5]. To confirm the success of the cloning process, the plasmids were isolated and sent for sequencing (*Eurofins*, Hamburg, BL, Germany).

Expression and purification

E. coli competent cells were transformed with recombinant plasmids for each protein and selected on Lysogenic Broth (LB) plates containing 50 μg mL^{-1} ampicillin. For each protein, two plates were prepared, one for each vector used. Single colonies generated by the transformation with the pCRE vector were collected and transferred into a test tube with 5 mL LB medium and 50 μg mL^{-1} ampicillin. The culture was incubated overnight to be used as a pre-inoculum. Subsequently, cells from the pre-inoculum were transferred into 200 mL of Terrific Broth (TB) medium containing 50 μg mL^{-1} ampicillin and incubated at 37 ºC with agitation at 135 rpm. Protein expression was induced by the addition of *L*-arabinose (0.02% v/v) when OD_{600} reached 0.6-0.8. The cells were then incubated at

24 ºC for 14-16 hours, while shaking at 135 rpm. After this period, cells were harvested by centrifugation (4420 x g, 20 minutes, 4 ºC - *Beckman-Coulter* centrifuge), the supernatant was discarded and the pellet was resuspended in 15 mL lysis buffer (50 mM Tris-HCl pH 7.5, 150 mM NaCl, 1 µg mL^{-1} DNAse, 0.1 mM phenylmethylsulphonyl fluoride). Cell disruption was achieved by ultrasonic treatment (5 sec on and 7 sec off for 10 minutes, 70% amplitude) and the resulting extract was clarified by centrifugation (8100 x g, 40 minutes, 4 °C – *Eppendorf* centrifuge).
The His-tagged proteins were purified using 3 mL HisTrap Ni-Sepharose HP columns (*GE Healthcare Lifesciences*, Boston, MS, USA). The column was equilibrated with 3 column volumes (CVs) of 50 mM Tris-HCl, 100 mM NaCl, pH 7.5. The cleared cell extract was loaded on the column after using a microfilter (Ø = 0.2 µm) in order to remove small precipitates. To enhance the interaction between the proteins and the nickel, the column was rotated for 1 hour at 4 ºC. Subsequently, the column was washed with 3 CVs of 50 mM Tris-HCl (pH 7.5) containing 5 mM imidazole. Following washing, the protein was eluted with 50 mM Tris-HCl, 500 mM imidazole (pH = 7.5). The collected volume of the yellow-colored enzyme fraction was 2.5 mL. The purified protein sample was desalted using a PD10 desalting column (*Cytiva* - *Global Life Sciences Solutions*, Morrisville, NC, USA) with 50 mM Tris-HCl (pH 7.5). The purified proteins were then analyzed for purity by SDS-PAGE (Figure S2) revealing that all enzymes have a molecular weight of approximately 85 kDa. Aliquots of purified proteins were frozen using liquid nitrogen and stored at −70 °C until further use.
For expression SUMO-fused ERs, expression and purification procedures were carried out as described above, with one difference: the expression temperature was set to 17 °C. Only PsOYE2 and PsOYE5 were obtained as soluble and flavin-containing protein. Aliquots of purified proteins were promptly frozen using liquid nitrogen and stored at −70 °C for future use.

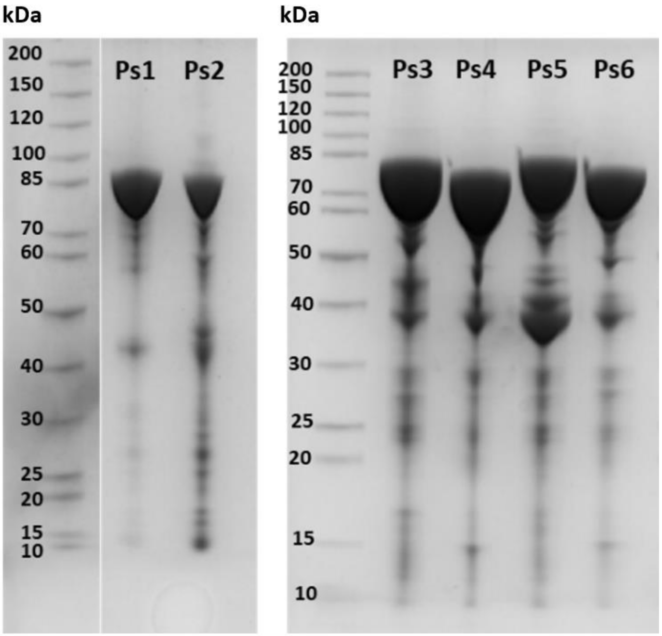

Figure S2. SDS-PAGE analysis of purified enzymes. The first lane shows the protein ladder and the lanes the enzymes in the following order: PsOYE1, PsOYE2, PsOYE3, PsOYE4, PsOYE5 and PsOYE6.

Spectral analysis

Absorbance spectra were acquired using a UV-spectrophotometer (*Jasco* V-650/660/730, Tokyo, Japan) for both the native and denatured proteins (Figure S3). Absorbance measurements were conducted in the wavelength range of 300 to 600 nm at 25 °C. Each protein was denatured by heating the sample at 95 ºC for 20 minutes and then, samples were centrifuged. The absorbance spectrum of the denatured protein sample was used to determine the molar extinction coefficient of each flavoprotein based on the known molar extinction coefficient of FMN (ε_{446} = 12.2 mM cm^{-1}). The maximum absorbance observed for all proteins, along with their extinction coefficients (estimated by comparison with the known molar extinction coefficient of FMN) and yield, is presented in Table S1.

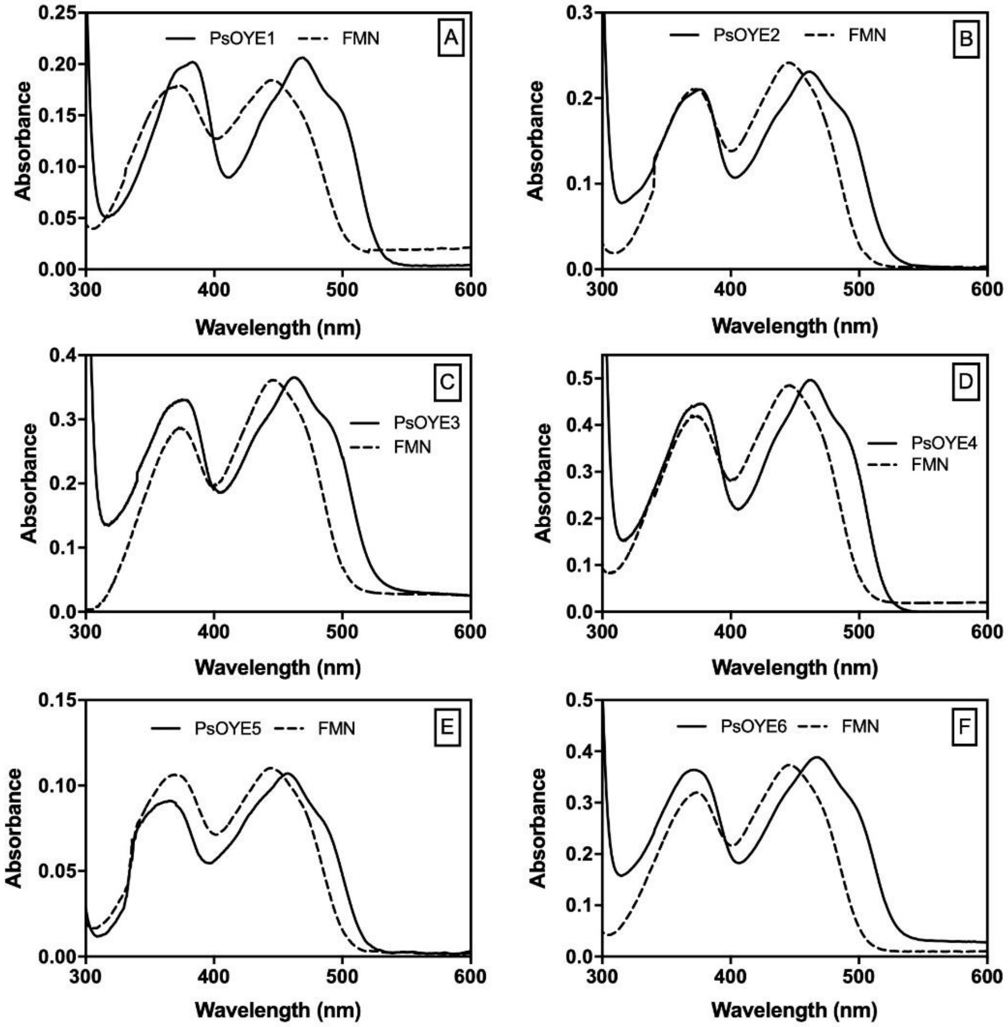

Figure S3. Absorbance spectra for PsOYE1 (**A**), PsOYE2 (**B**), PsOYE3 (**C**), PsOYE4 (**D**), PsOYE5 (**E**) and PsOYE6 (**F**), native (–) and denatured (--).

Table S2. Molar extinction coefficients and yields of purified proteins.

Enzyme	Absorbance maxima (nm)	Extinction coefficient (mM^{-1} cm^{-1})	Yield (mg L^{-1})
**PsOYE1**	383, 469	14.3	61
**PsOYE2**	376, 461	12.0	53
**PsOYE3**	375, 462	13.0	61
**PsOYE4**	376, 462	13.4	153
**PsOYE5**	369, 456	11.8	29
**PsOYE6**	371, 467	12.0	121

Enzyme activity analysis

Enzyme activity was measured by monitoring the consumption of NADH or NADPH at 340 or 365 nm using a spectrophotometer. Measurements were performed at 25 ºC in 100 µL reactions using 50 mM Tris-HCl (pH 7.5) as buffer. For substrate screening, 1.0 mM of the test substrate and 30 nM of enzyme was used. Activities are expressed in units, where 1 unit (U) represents the amount of enzyme required to convert 1 µM mol NAD(P)H in 1 minute. The following compounds were tested as substrates: tiglic aldehyde, *R*-carvone, cinnamic acid, cyclohex-2-en-1-one, maleimide, methyl cinnamate, *p*-benzoquinone, and *trans*-4-phenyl-but-3-en-2-one. The ability of the reductases to utilize dioxygen as electron acceptor, essentially acting a NAD(P)H oxidase, was also investigated. When such NAD(P)H consumption activity was observed in absence of an ene substrate, the reported ene reduction activity was corrected for this futile oxidase reactivity. Activity screening was performed with both nicotinamide cofactors, NADH and NADPH, to establish cofactor preference.

For a more detailed kinetic analysis, *p*-benzoquinone were selected and used to determine the steady state kinetic parameters, K_M and k_{cat}. For this, the consumption of NADPH was monitored at 340 nm in a 100 µL reaction in 50 mM Tris-HCl (pH 7.5) containing 100 µM NADPH, 30 nM enzyme, and 1.0 mM *p*-benzoquinone. For determining the K_M, NADPH, the nicotinamide concentration was varied (25 - 600 µM) while using *p*-benzoquinone at a fixed concentration (1.0 mM). The data were fitted using the Michaelis-Menten equation in the program *Graph-Pad Prism* v. 6.07 (*GraphPad Software*, San Diego, CA, USA). The Michaelis-Menten curves obtained are presented in Figure S4 and S5. All the measurements were performed in triplicate.

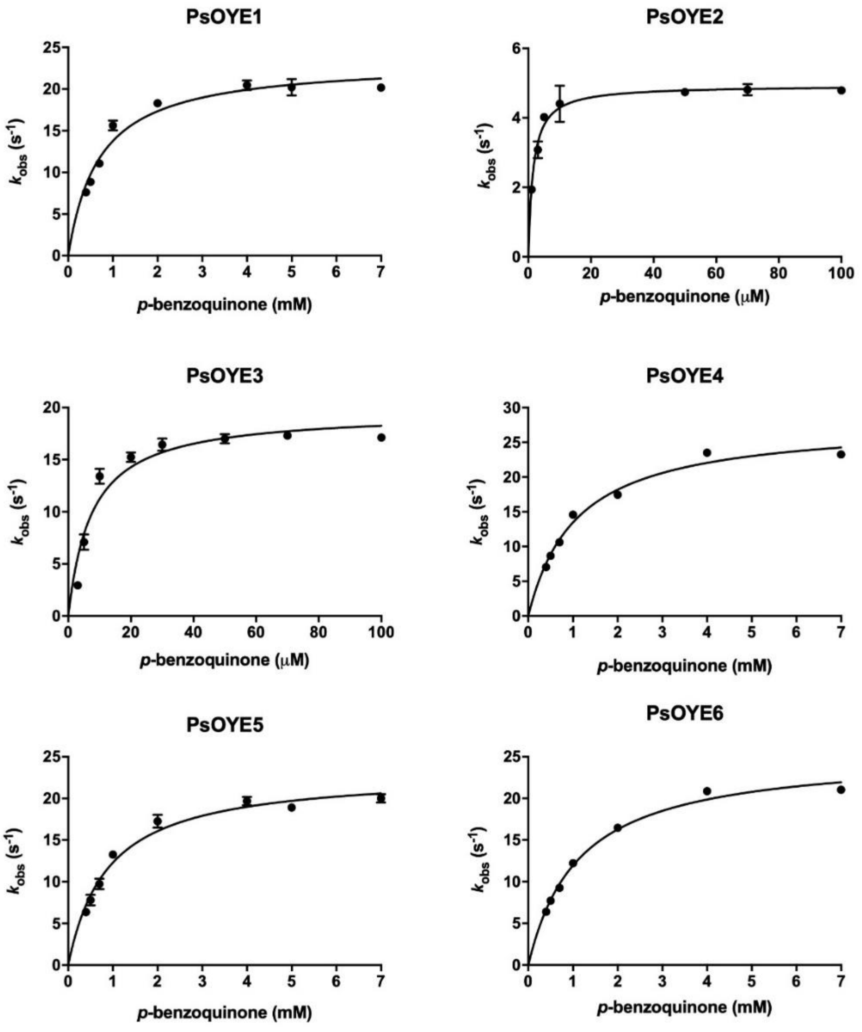

Figure S4. Michaelis-Menten curves obtained by the software GraphPad Prism for measurement of kinetic parameters (K_M and k_{cat}) for the enzymes PsOYE1, PsOYE2, PsOYE3, PsOYE4, PsOYE5 and PsOYE6.for *p*-benzoquinone The kinetic parameters were calculated by analyzing the consumption of NADPH at 340 nm.

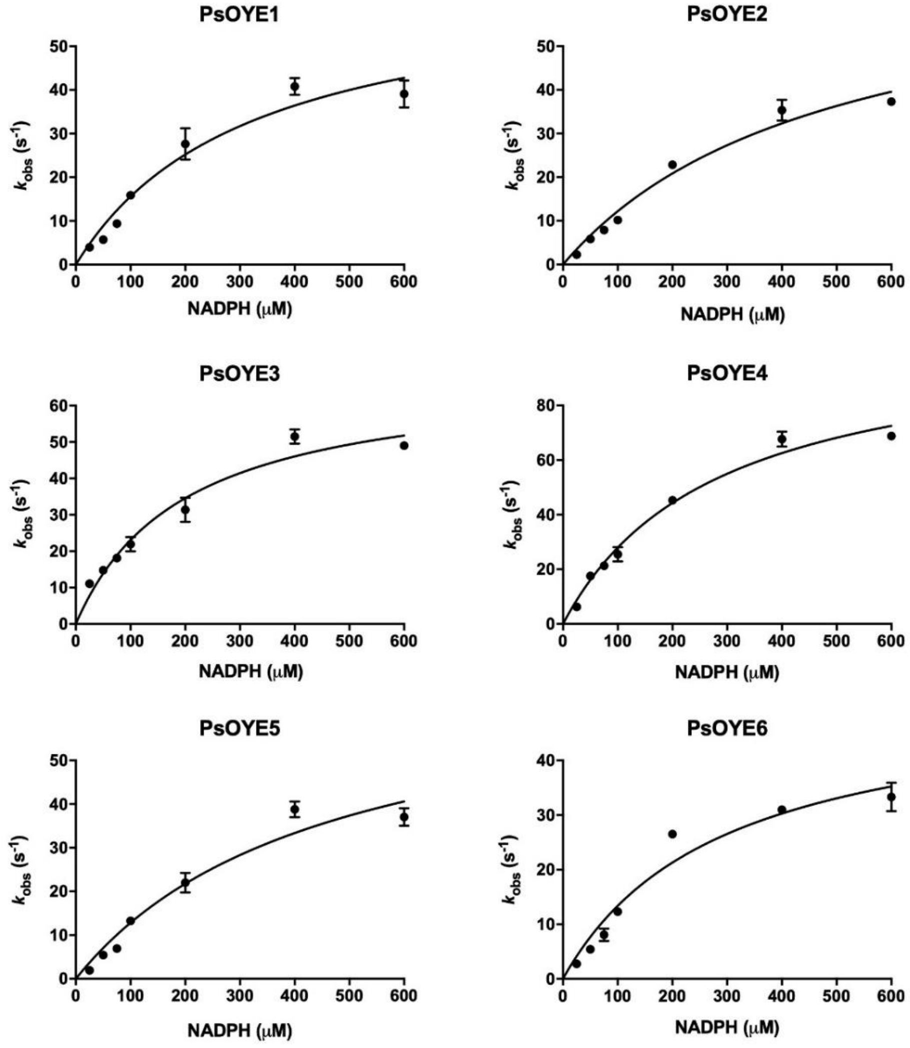

Figure S5. Michaelis-Menten curves obtained by the software GraphPad Prism for measurement of kinetic parameters (K_M and k_{cat}) for the enzymes PsOYE1, PsOYE2, PsOYE3, PsOYE4, PsOYE5 and PsOYE6 for NADPH using *p*-benzoquinone in fixed concentration (1mM). The kinetic parameters were calculated by analyzing the consumption of NADPH at 340nm.

The pH optima for activity were determined using different buffers: 50 mM acetate buffer for pH 4.0 and 5.0, 50 mM KP_i buffer for pH 6.0 and 7.0, and 50 mM Tris-HCl buffer for pH 8.0 and 9.0. For each buffer, the consumption of NADPH (0.10 mM) was monitored at 340 nm using *p*-benzoquinone (1.0 mM) as the substrate. All the measurements were performed in triplicate and the curves obtained are shown in Figure S6.

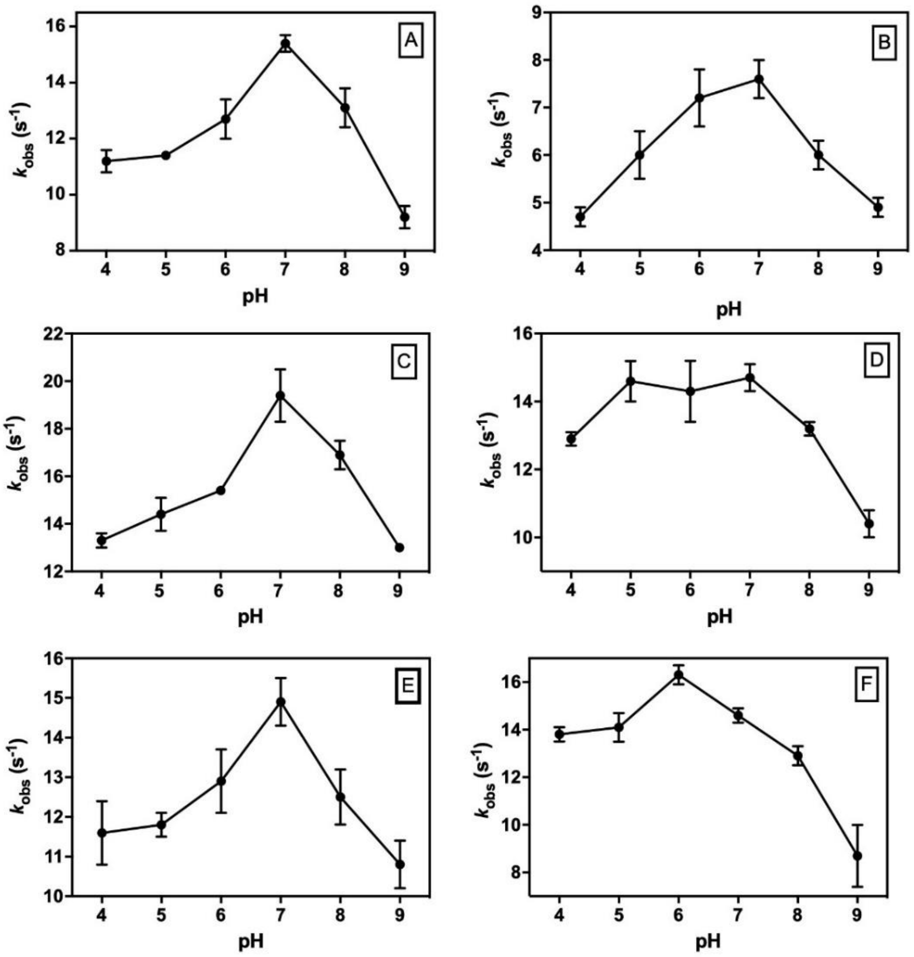

Figure S6. pH optima for activity for PsOYE1 (**A**), PsOYE2 (**B**), PsOYE3 (**C**), PsOYE4 (**D**), PsOYE5 (**E**) and PsOYE6 (**F**).

Analysis of thermostability

For all enzymes, the apparent unfolding temperatures (T_m) were determined in 50 mM Tris-HCl at pH 7.5. To assess thermostability, the ThermoFAD [7] procedure was utilized, relying on the fluorescence of the bound flavin cofactor. Sample volumes of 20 µL were used, each containing 10 µM enzyme. The measurements were conducted in a RT–PCR machine (CFX96 – *Bio-Rad*, Hercule, CA, USA) using SYBR fluorescence settings (excitation filter: 450-490 nm; emission filter: 515-530 nm). The fluorescence was recorded as the temperature increased from 25 ºC until 90 ºC, with a heating rate of 0.5°C per 30 seconds. Each reported T_m is based on two measurements. For enzyme PsOYE5, the Thermofluor® [8] procedure was also performed. The same procedure was repeated, but in contrast to ThermoFAD, SYPRO Orange dye was added into the samples before analysis and FRET fluorescence settings (excitation filter: 450-490 nm; emission filter: 560-590 nm) were used to monitor unfolding.

Crystallization and structure determination

For crystallography analysis, the His-tagged SUMO fusion protein was removed from PsOYE 2 and PsOYE 5 by adding SUMO protease and incubating at 4 °C overnight. The SDS gel analysis is presented in Figure S7, from which it was possible to see the proteins without SUMO with a molecular weight of approximately 40 kDa.

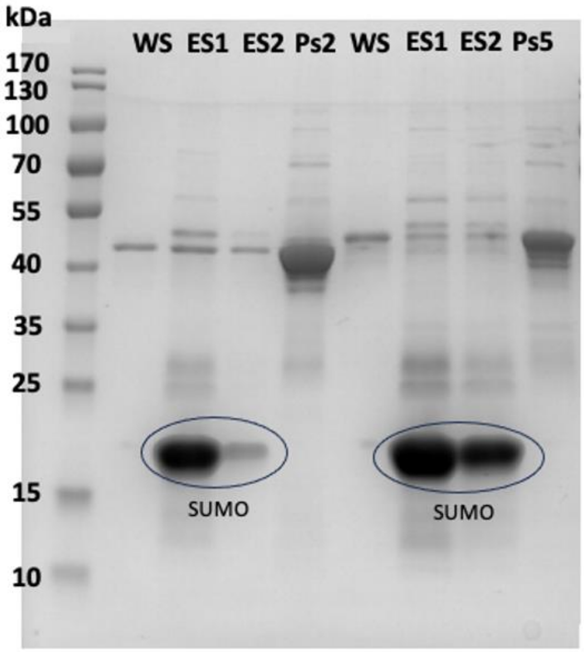

Figure S7. SDS-PAGE analysis of the purified enzymes (PsOYE2 and PsOYE5) after removing SUMO. The first lane shows the protein ladder, and the followings lanes correspond to washing solution (WS), elution solution 1 and 2 (ES1/2) and PsOYE2 and PsOYE5. The SUMO protein in the gel was circled.

Enzymes obtained after SUMO cleavage were further purified by gel filtration using a Superdex 200 HR10/30 column (*Cytiva*) and equilibrated with 150 mM NaCl, 20 mM HEPES (pH 7.3). Purification was carried out on an *Akta*® explorer system with wavelengths set at 280, 254 and 460 nm. Yellow fractions were pooled and concentrated to 10 mg.mL^{-1} using an Ultracel-30K filter unit (*Millipore*). Dynamic light scattering (DLS) experiments were performed using a DynaPro MS800TC instrument (*Wyatt Technology Corporation,* Santa Barbara, CA, USA) at 294 K. DLS data were processed and analyzed with Dynamics software.
The following steps were exclusively applied only for PsOYE2, which could be successfully purified and crystallized. Initial sitting-drop crystallization screening was performed using a Mosquito crystallization robot (*STP Labtech*) in 96-well MRC2 plates (Molecular Dimensions). Several commercially available screening solutions were tested. Crystals were successfully grown from a solution containing 0.16 M Ca-acetate, 0.08 M Na-cacodylate pH 6.5, 14.4% PEG8000 and 20% glycerol. X-ray diffraction data were recorded at the MASSIF-1 beamline at the ESRF (Grenoble, France) [9]. Data processing, scaling, and merging were carried out with XDS [10], imosflm [11] and autoPROC [12] followed by AIMLESS [13].
The structure of the PsOYE2 was determined by the molecular replacement method using Phaser [14] with an *Alphafold2* model of the ER. Model building and structure analysis were performed by the program COOT [15], while refinement was carried out using REFMAC5 [16] and autoPROC-isotropic [17]. In later stages, phenix.refine [18] was employed, with hemihedral twin refinement using the twin operator -l, -k, -h and twin fraction 0.46. The quality of the model was analyzed with PDB_REDO [19] and MolProbity [20]. *PyMOL* was used for figure preparation. Data collection statistics and refinement details were recorded in Table S3. Atomic coordinates and experimental structure factor amplitudes were deposited in the Protein Data Bank (PDB) number 8S4P.
The structure of PSOYE2 was aligned with the sequences of proteins with the closest homologous structures: AnOYE8 from *Aspergillus niger* (PDB: 7QFX) and BfOYE4 from *Botryotinia fuckeliana* (PDB: 7BLF), and it is presented in Figure S8.

Table S3. Data collection and refinement statistics for PsOYE2.

**PDB code**	**8S4P**
**Space group**	P21
**Resolution range (Å)**	86.9 -2.29 (2.33 - 2.29)
**Cell dimensions (Å)**	a = 107.48, b = 175.3, c = 107.53<br>α = 90.0, β = 111.5, γ = 90.0
**Unique reflections**	164580 (8106)
**Completeness (%)**	99.1 (98.5)
**Multiplicity**	6.9 (6.9)
**$CC_{1/2}$**	0.991 (0.724)
**Overall I/σ (I)**	8.3 (2.2)
**Rmerge (%)**	0.204 (0.958)
**Rpim (%)**	0.084 (0.391)
***R/ Rfree* (%)**	20.1 / 27.5
**N° of protein residues**	3276
**N° of FMN molecules**	8
**N° of water molecules**	1085
**N° of Ca^{2+} ions**	6
**N° of Cl^{-} ions**	7
**Protein B value ($Å^2$)**	24.7
**FMN B values ($Å^2$)**	26.9
**Ramachandran outliers (%)**	0.1
**Ramachandran favored (%)**	96.2
**Clash scores**	12.0
**Molprobity score**	2.35
**RMSD bond length (Å)**	0.008
**RMSD bond angles (°)**	1.02
**PDB code**	8S4P
**Space group**	P21
**Resolution range (Å)**	86.9 -2.29 (2.33 - 2.29)

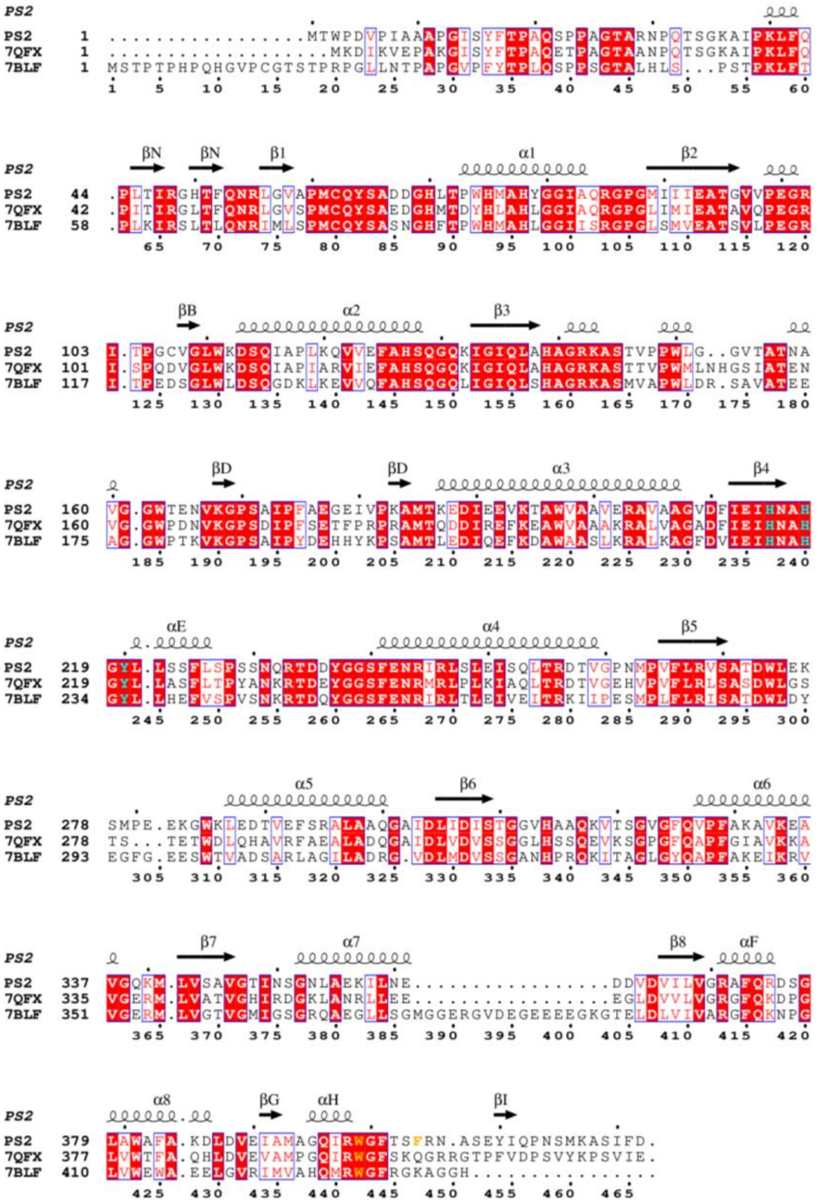

Figure S8. Structure-based sequence alignment of PsOYE2 and 7BLF (OYE4 from *Botryotinia fuckeliana* (BfOYE4)) and 7QFX (OYE8 from *Aspergillus niger* (AnOYE8)). The alignment was made with SSM (ebi.ac.uk/msd-srv/ssm/). The secondary structure elements (top line) are from the crystal structure of PsOYE2. Identical residues have a red background, similar residues have a red color. The active site residues are in cyan and Phe^{404} and Trp^{399} are in orange. The figure was created with *ESPrip*

Bioconversion

PsOYE3, PsOYE4, and PSOYE5 were used to reduce *R*-carvone in 100 mM Tris-HCl (pH 7.5) with 10 mM substrate (1% DMSO), 0.20 mM NADPH, 30 nM enzyme, and 20 mM sodium phosphite. The reaction mixture (1 mL) was incubated at 25 °C for 12 h with agitation (400 rpm). Termination involved adding ethyl acetate (1:1, v/v) for product extraction followed by centrifugation. Ethyl acetate was added again to the aqueous phase (1:1, v/v) for thorough extraction. The combined organic phases were dehydrated with Na2SO4. Mesitylene (0.02% v/v) was included as an internal standard.

The conversion and *d.e.* were determined by GC-MS using a HP-5MS column (30 m × 0.25 mm × 0.25 µm, Agilent). A 1 µL sample was injected with helium as the carrier gas (flow rate: 1.7 ml/min) using a 10:1 split ratio, and injection was performed at 300 °C. The temperature program included a ramp of 20 °C/min from 40 °C to 325 °C, followed by a 5-minute hold at 325 °C. The mass spectrometer operated as the detector, utilizing electron ionization (EI) at 70 eV, and maintaining an ion source temperature of 200 °C. Fragment

ions were detected within the 35–500 Da range. Retention times were: 8.6 min for mesitylene (internal standard), 11.3 min for *R*-carvone, 10.9 min for 2*R*,5*R*-dihydrocarvone and 11.0 min for (2*S*,5*R*)-dihydrocarvone. The chromatograms obtained are presented in Figure S9.

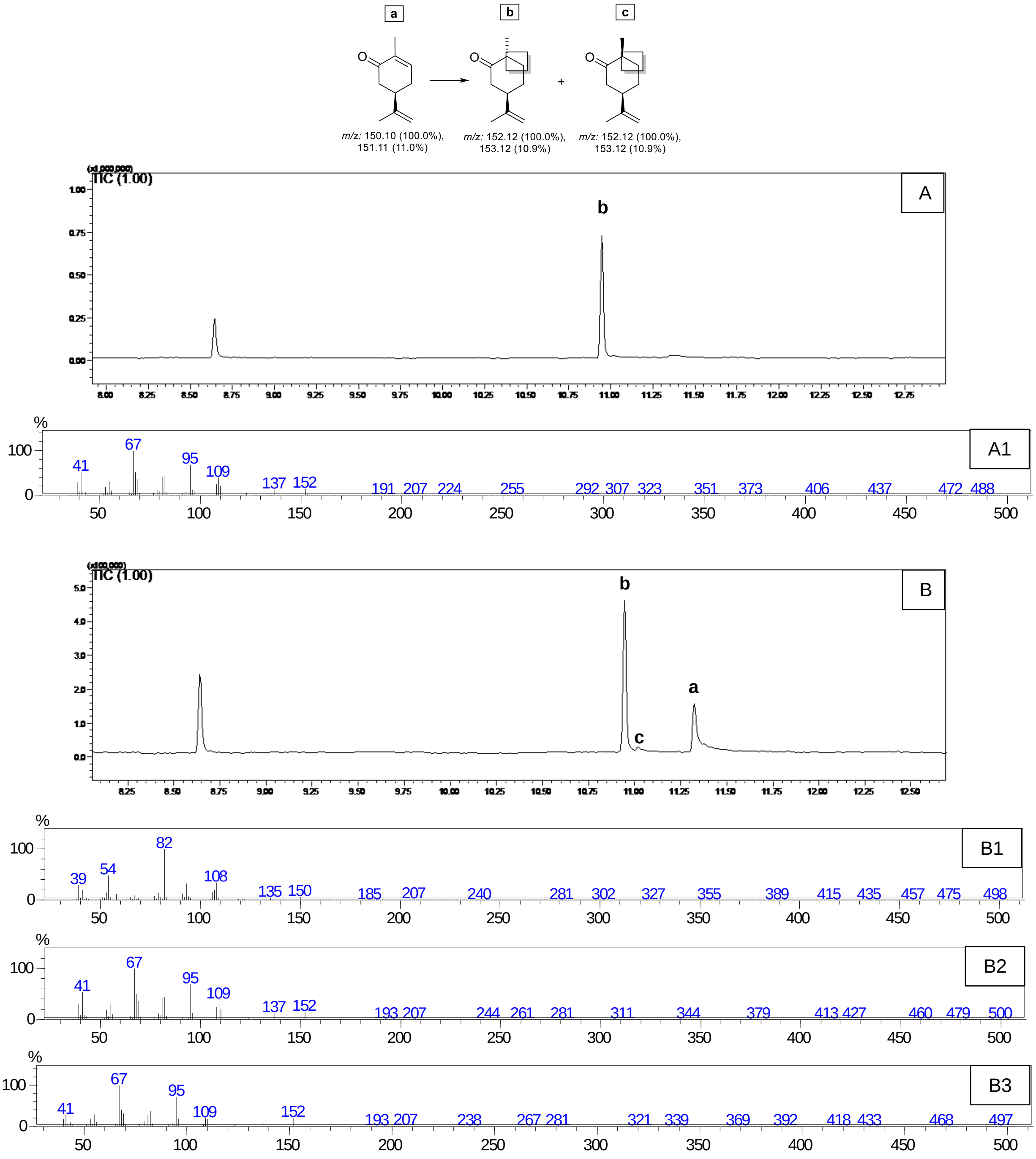

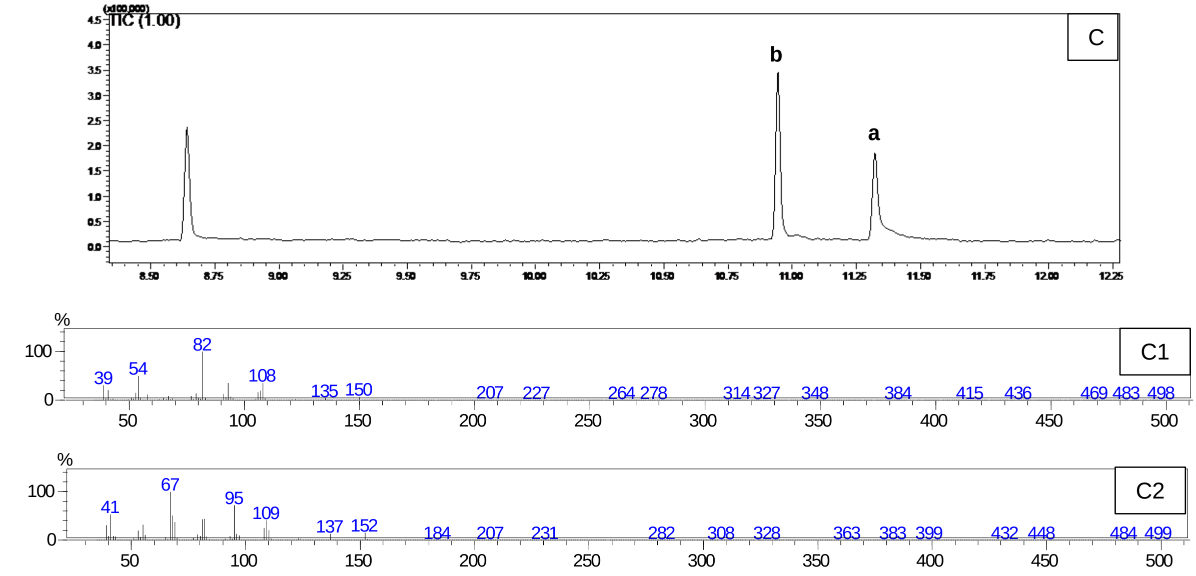

Figure S9. Chromatograms and mass spectra obtained during the bioconversion of *R*-carvone (**a**) into (2*R*,5*R*)-dihydrocarvone (**b**) and (2*S*,5*R*)-dihydrocarvone (**c**) are presented as follows: Chromatogram (**A**) for the reaction with PsOYE3 and mass spectrum related to the peak of compound **b** (**A1**); chromatogram (**B**) for the reaction with PsOYE4 and mass spectra for the peaks of the compounds: **a** (**B1**), **b** (**B2**), and **c** (**B3**); chromatogram (**C**) for the reaction with PsOYE5 and mass spectra for the peaks of the compounds: **a** (**C1**), and **b** (**C2**). Mesitylene was used as an internal standard (RT = 8.6 min).

The stereochemical configuration of the products was determined by comparison with the chromatogram of a commercial dihydrocarvone standard, which predominantly contains the (*R,R*) stereoisomer (Figure S10).The chromatogram for commercial dyhidrocarvone was obtained by GC-MS (Shimadzu GC2010 Plus, MS2010 Plus) in electron ionization mode (70 eV). A 1.0 µL sample was injected into a DB5 column (30 m × 0.25 mm × 0.25 µm) with injector and interface temperatures of 250 °C and 270 °C. The oven temperature was held at 90 °C for 4 min, then ramped to 280 °C at 10 °C/min (held for 5 min), followed by an increase to 300 °C (held for 10 min). Total analysis time was 40 min. Helium (0.75 mL/min) was used as carrier gas with a split ratio of 20:1. Fragment ions were detected between 40–550 Da.

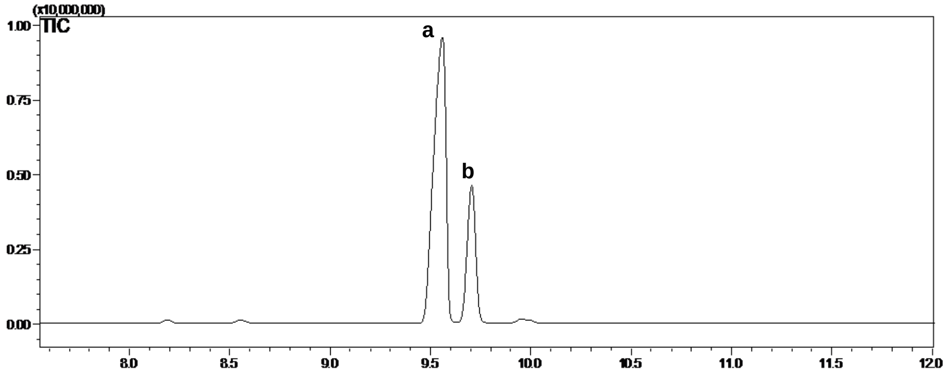

Figure S10. Chromatogram illustrating the composition of commercial dihydrocarvone, consisting of a mixture of isomers with varying concentrations. The concentration of *R*,*R*-dihydrocarvone (**a**) is higher than that of *S*,*R*-dihydrocarvone (**b**).

References

[1] S. Böhmer, C. Marx, Á. Gómez-Baraibar, M. M. Nowaczyk, D. Tischler, A. Hemschemeier, T. Happe, Algal Res. 2020, 50, 101970.

[2] Tamura, K., Stecher, G., Kumar, S., Mol. Biol. Evol. **2021**, 38, 3022–3027.

[3] D. T. Jones, W. R. Taylor, J. M. Thornton, Comput. Appl. Biosci. **1992**, 8, 275–282.

[4] E. Zuckerkandl, L. Pauling in Evolving Genes and Proteins (Eds.: V. Bryson, H. J. Vogel), Academic Press, 1965, pp. 97–166.

[5] D. E. Torres Pazmiño, A. Riebel, J. De Lange, F. Rudroff, M. D. Mihovilovic, M. W. Fraaije, ChemBioChem **2009**, 10, 2595–2598.

[6] C. Engler, R. Kandzia, S. Marillonnet, PLoS ONE **2008**, 3, e3647.

[7] F. Forneris, R. Orru, D. Bonivento, L. R. Chiarelli, A. Mattevi, FEBS J. **2009**, 276, 2833–2840.

[8] M. D. Cummings, M. A. Farnum, M. I. Nelen, SLAS Discov. **2006**, 11, 854–863.

[9] M. W. Bowler, D. Nurizzo, R. Barrett, A. Beteva, M. Bodin, H. Caserotto, S. Delagenière, F. Dobias, D. Flot, T. Giraud, N. Guichard, M. Guijarro, M. Lentini, G. A. Leonard, S. McSweeney, M. Oskarsson, W. Schmidt, A. Snigirev, D. von Stetten, C. Mueller-Dieckmann, J. Synchrotron Radiat. **2015**, 22, 1540–1547.

[10] W. Kabsch, Acta Crystallogr. Sect. D Biol. Crystallogr. **2010**, 66, 125–132.

[11] T. G. G. Battye, L. Kontogiannis, O. Johnson, H. R. Powell, A. G. W. Leslie, Acta Crystallogr. Sect. D Biol. Crystallogr. **2011**, 67, 271–281.

[12] C. Vonrhein, C. Flensburg, P. Keller, A. Sharff, O. Smart, W. Paciorek, T. Womack, G. Bricogne, Acta Crystallogr. Sect. D Biol. Crystallogr. **2011**, 67, 293–302.

[13] P. R. Evans, G. N. Murshudov, Acta Crystallogr. Sect. D Biol. Crystallogr. **2013**, 69, 1204–1214.

[14] A. J. McCoy, R. W. Grosse-Kunstleve, P. D. Adams, M. D. Winn, L. C. Storoni, R. J. Read, J. Appl. Crystallogr. **2007**, 40, 658–674.

[15] P. Emsley, B. Lohkamp, W. G. Scott, K. Cowtan, Acta Crystallogr. Sect. D Biol. Crystallogr. **2010**, 66, 486–501.

[16] G. N. Murshudov, P. Skubák, A. A. Lebedev, N. S. Pannu, R. A. Steiner, R. A. Nicholls, M. D. Winn, F. Long, A. A. Vagin, Acta Crystallogr. Sect. D Biol. Crystallogr. **2011**, 67, 355–367.

[17] P. V. Afonine, R. W. Grosse-Kunstleve, N. Echols, J. J. Headd, N. W. Moriarty, M. Mustyakimov, T. C. Terwilliger, A. Urzhumtsev, P. H. Zwart, P. D. Adams, Acta Crystallogr. Sect. D Biol. Crystallogr. **2012**, 68, 352–367.

[18] V. B. Chen, W. B. Arendall, J. J. Headd, D. A. Keedy, R. M. Immormino, G. J. Kapral, L. W. Murray, J. S. Richardson, D. C. Richardson, Acta Crystallogr. Sect. D Biol. Crystallogr. **2010**, 66, 12–21.

[19] R. P. Joosten, F. Long, G. N. Murshudov, A. Perrakis, IUCrJ **2014**, 1, 213–22
